# *E. coli* catheter-associated urinary tract infections are associated with distinctive virulence and biofilm gene determinants

**DOI:** 10.1172/jci.insight.161461

**Published:** 2023-01-24

**Authors:** Zongsen Zou, Robert F. Potter, William H. McCoy, John A. Wildenthal, George L. Katumba, Peter J. Mucha, Gautam Dantas, Jeffrey P. Henderson

**Affiliations:** 1 Center for Women’s Infectious Diseases Research,; 2 Department of Internal Medicine, Division of Infectious Diseases,; 3 The Edison Family Center for Genome Sciences and Systems Biology,; 4 Department of Pathology and Immunology, and; 5 Department of Internal Medicine, Division of Dermatology, Washington University School of Medicine, St. Louis, Missouri, USA.; 6 Department of Mathematics, Dartmouth College, Hanover, New Hampshire, USA.; 7 Department of Molecular Microbiology, Washington University School of Medicine, St. Louis, Missouri, USA.; 8 Department of Biomedical Engineering, Washington University in St. Louis, Missouri, USA.

**Keywords:** Infectious disease, Microbiology, Bacterial infections, UTI/pyelonephritis, Urology

## Abstract

Urinary catheterization facilitates urinary tract colonization by *E. coli* and increases infection risk. Here, we aimed to identify strain-specific characteristics associated with the transition from colonization to infection in catheterized patients. In a single-site study population, we compared *E. coli* isolates from patients with catheter-associated asymptomatic bacteriuria (CAASB) to those with catheter-associated urinary tract infection (CAUTI). CAUTI isolates were dominated by a phylotype B2 subclade containing the multidrug-resistant ST131 lineage relative to CAASB isolates, which were phylogenetically more diverse. A distinctive combination of virulence-associated genes was present in the CAUTI-associated B2 subclade. Catheter-associated biofilm formation was widespread among isolates and did not distinguish CAUTI from CAASB strains. Preincubation with CAASB strains could inhibit catheter colonization by multiple ST131 CAUTI isolates. Comparative genomic analysis identified a group of variable genes associated with high catheter biofilm formation present in both CAUTI and CAASB strains. Among these, ferric citrate transport (Fec) system genes were experimentally associated with enhanced catheter biofilm formation using reporter and *fecA* deletion strains. These results are consistent with a variable role for catheter biofilm formation in promoting CAUTI by ST131-like strains or resisting CAUTI by lower-risk strains that engage in niche exclusion.

## Introduction

Catheter-associated urinary tract infections (CAUTI) are among the most common nosocomial infections, with over 1 million cases annually in the United States (1–3). Accurate diagnosis and effective treatment of CAUTI and ureteral stent–associated infections can be challenging (4). Bacteriuria alone is an insufficient criterion to establish a CAUTI diagnosis, which also requires attributable patient signs or symptoms such as suprapubic tenderness, flank pain, or fever (5, 6). For patients with catheter-associated asymptomatic bacteriuria (CAASB) who are at low risk of serious infection, antibiotics are not recommended (7). When clinical status plausibly masks symptoms or symptoms are not clearly attributable to the urinary tract, physicians must weigh the risk of progressive infection against the risks of catheter or device removal and inappropriate antibiotic therapy. In this context, professional society guidelines have long noted a need to better discriminate CAASB from CAUTI and to predict a patient’s risk for progression to CAUTI (8, 9).

The pathogenic potential of any given bacterial strain is a function of both host and bacterial characteristics (10). In the urinary tract, the presence of a catheter or stent is an especially influential host characteristic, conferring a well-recognized predisposition to bacterial colonization and infection (2, 3, 11). By affecting urinary flow, providing an abiotic surface for bacterial adherence, and changing the local epithelium (12–14), these devices are associated with a distinctive pathophysiology. The ability of *E*. *coli* to form biofilms is generally regarded as an important virulence characteristic in catheterized patients (15, 16). Biofilms are adherent bacterial communities enmeshed in an extracellular polymeric substance (EPS) matrix that form in response to specific environmental cues, permitting a resident bacterial population to expand and persist in the urinary tract lumen. In the laboratory, a single *E*. *coli* strain can form qualitatively and quantitatively distinctive biofilms, depending upon media composition, temperature, and flow conditions (17, 18).

### E.

*coli* is the predominant bacterial species associated with asymptomatic bacteriuria, uncomplicated UTI, CAASB, and CAUTI (19). Unlike enteric pathotypes, there is no definitive genetic signature of a “uropathogenic” *E*. *coli* strain. Studies have identified *E*. *coli* characteristics that are common in the setting of infection but none that are definitive of a uropathogenic pathotype, consistent with the view that the uropathogenic potential of *E*. *coli* is multifactorial in nature (20). The virulence factors (VFs) identified to date have mostly been studied in uncomplicated UTIs, are functionally diverse, and may contribute to pathogenic potential differently in catheterized patients. Rather than achieving a strict monogenic definition for uropathogenic *E*. *coli,* data from uncomplicated UTIs have been most consistent with a probabilistic and combinatorial relationship between virulence determinants and disease (19, 21).

In the present study, we sought to compare *E*. *coli* strain characteristics between patients with CAUTI and CAASB. Intrinsically, asymptomatic isolates are more difficult to find in the clinical setting, as they must be drawn in the absence of attributable symptoms. We were able to identify CAASB isolates, along with CAUTI isolates, from a previously described observational cohort study (5, 6). Comparisons were based on whole-genome sequencing analyses and quantitative biofilm phenotyping using a simulated catheter biofilm system. Comparative genomic analyses were used in conjunction with network community analysis to identify gene combinations associated with infection and catheter biofilm formation. CAUTI strains were associated with sequence type 131 (ST131), a lineage with high antibiotic resistance and distinctive virulence genes. Using a competitive catheter biofilm assay, we identified a subset of CAASB isolates capable of preventing colonization by CAUTI-associated, ST131 isolates. Multiple gene communities were associated with high catheter biofilm formation from comparative genomic analysis. Finally, we used a transcriptional reporter and a reverse bacterial genetic approach to functionally connect the ferric citrate uptake system (Fec), which exhibited the strongest relationship with catheter biofilm in genomic comparison analyses, to *E*. *coli* biofilm formation.

## Results

### E. coli isolates.

To compare *E*. *coli* strains associated with CAUTI or CAASB in hospitalized patients, we identified 62 catheter-associated isolates (18.4%) from a previously described collection of 337 urinary isolates from patients at Barnes-Jewish Hospital/Washington University Medical Center between August 1, 2009, and July 31, 2010 (5, 6). Of these 62 isolates, 12 met symptom criteria for CAUTI (concurrent fever, body temperature [T] > 38°C), and 16 met criteria for CAASB (lack of fever or other clinical symptoms). As an additional comparator group, 13 *E*. *coli* isolates corresponding to asymptomatic rectal colonization were collected by rectal swabs from healthy, adult volunteers at Barnes-Jewish Hospital/Washington University Medical Center from 2014 to 2015, designated as rectal colonizers (RC) (Table 1). In total, 41 *E*. *coli* isolates were collected for this study, with each isolate from a unique catheterized patient or healthy volunteer. CAUTI and CAASB participants were of similar age and BMI but exhibited a significant difference in results between males and females (*P* = 0.0093). Bacteriuric inpatient participants were older than nonhospitalized asymptomatic RC participants (*P* = 0.0309), typical of inpatients in the United States (22). Moreover, *E*. *coli* strains isolated from urinary bacteriuria were identified with higher trimethoprim/sulfamethoxazole (TMP/SX) and quinolone resistance in clinical laboratory tests (*P* < 0.01), consistent with multidrug resistance facilitating urinary colonization in catheterized patients.

### Phylogenomic analysis.

**Figure 1:**
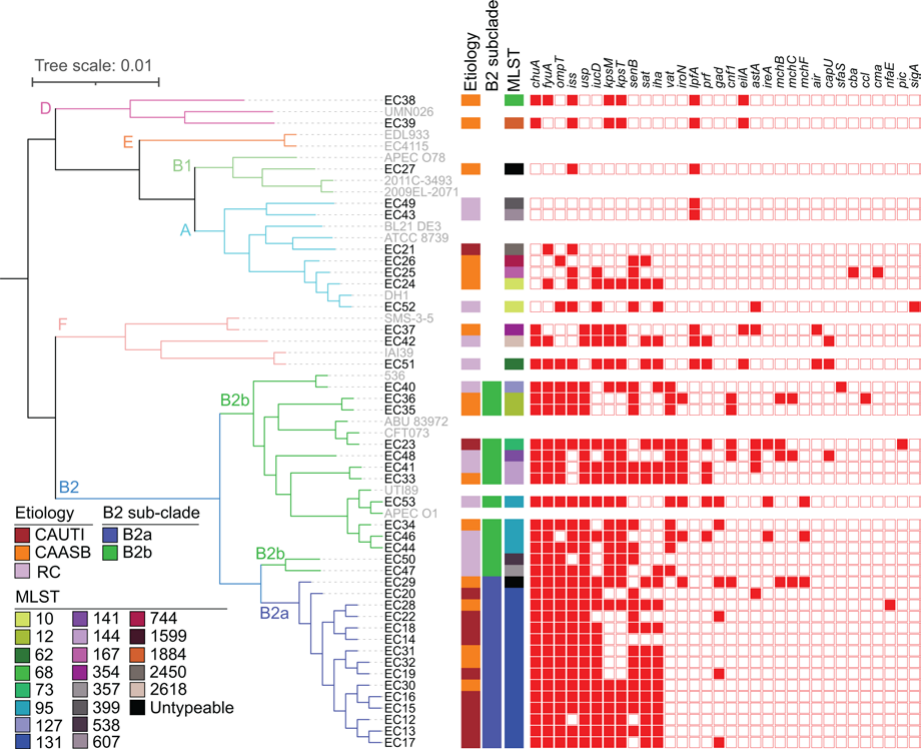
Phylogenetic distribution of 41 clinical *E*. *coli* isolates. Core-genome alignment was constructed into a maximum likelihood tree with raxML and viewed in iTOL, with 6 phylotypes identified. In silico multilocus sequence types (MLST) were identified using BLASTN to the *E*. *coli* MLST database, with 21 STs identified. Virulence factors (VFs) were annotated in the *E*. *coli* draft genomes using VirulenceFinder v1.5 and blastp to previously described genes, with 32 VFs identified. The strain names of *E*. *coli* sequenced in this study were in black and reference *E*. *coli* strains were in gray.

We characterized the genome composition of all 41 *E*. *coli* isolates using a whole-genome sequencing approach. Of the 15,993 genes identified in the pangenome of these isolates, 2,458 were identified in 100% of isolates; 3,014 in 98% (40/41) of isolates; and more than 4,030 in 50% or fewer isolates. Each isolate was recognized as genetically distinct by comparing their genome differences, without clonal pairs, demonstrated by pangenome sizes ranging from 4,320 to 5,983 genes (Supplemental Table 1; supplemental material available online with this article; https://doi.org/10.1172/jci.insight.161461DS1) as well as their phylogenetic differences (Figure 1). A maximum-likelihood tree in Figure 1 shows the similarities and differences in gene content between the isolates. This unsupervised hierarchical phylogenetic clustering divides isolates into 4 main clades corresponding to the canonical *E*. *coli* phylotypes B2, F, and D and a combination of A, B1, and E (23). Nearly all CAUTI isolates (11 of 12) belonged to phylotype B2, as is typical of extraintestinal *E*. *coli* (2, 3). CAASB strains were more broadly distributed among all detected phylotypes, with the exception of phylotype E. RC isolates were distributed among phylotypes A, B2, and F. Of note, CAUTI strains clustered at the extreme of the phylogenetic distribution, corresponding to a subclade within phylotype B2 (designated as B2a), that disproportionately contains CAUTI isolates when compared with other B2 strains (designated B2b) (10 of 15 versus 1 of 13; *P* = 0.0021, 2-tailed Fisher’s exact test). The B2a subclade consisted of 14 ST131 strains (24, 25), while the non-B2a isolates (designated as B2b) were more diverse and consisted of 8 STs (STs 12, 73, 95, 127, 141, 144, 357, and 538). The prototypical non-CAUTI model strains UTI89 and CFT073 were not associated with B2a (Figure 1). Sparse principal component analysis (sPCA) (26, 27) of B2 strain genome composition similarly distinguished B2a from B2b strains, with clear separation on the PC1 (31%) in the score plot (Supplemental Figure 1, A and B). Classification of these B2 subclades by logistic regression (LR) using PC1 values yielded a prediction accuracy of 1.0 (Supplemental Figure 1C; SD = 0) and an AUC of 1.0 (Supplemental Figure 1D; SD = 0) in 5-fold cross validation. The pangenome sizes of B2a subclade isolates ranged from 4,578 to 5,983 genes (Supplemental Table 2). Pairwise core-genome alignment comparison among B2a isolates identified 34–2,784 single-nucleotide polymorphisms (SNPs; Supplemental Data File 1). These results demonstrate genetic differences among B2a isolates that are inconsistent with clonal pairs. Together, these analyses identified robust, systematic differences in *E*. *coli* gene composition in the CAUTI-associated B2a subclade.

### Antibiotic resistance.

The ST131 isolates that dominate B2a are a globally emergent extraintestinal pathogenic *E*. *coli* lineage associated with multidrug resistance, most notably to the fluoroquinolone class of antibiotics (24, 25). To determine whether increased antibiotic resistance is associated with B2a, we assessed antibiotic resistance gene (ARG) content and phenotypic resistance reported by the clinical laboratory. ARGs against aminoglycosides, β-lactams, amphenicols, TMP/SX, macrolides/lincosamides/streptogramins (MLS), quinolones, and tetracyclines were identified from the genome assembly (Supplemental Table 3). Both phenotypic and genotypic fluoroquinolone resistance were more common in B2a than B2b strains (Table 2; *P* = 0.0001). Specific SNPs previously associated with fluoroquinolone resistance in H30 subclones of ST131 strains (*gyrA* D87N, S83L; *parC* E84V, S80I; *parE* I529L) were nearly ubiquitous in B2a isolates (Table 2; *P* = 0.0001). Moreover, B2a group strains exhibited higher frequencies of ARGs associated with TMP/SX, β-lactams, and aminoglycosides (Supplemental Table 3; *P* < 0.03). The high frequency of resistance genes, particularly those related to fluoroquinolones, is consistent with previously described ST131 isolates (24, 25).

### VF content of CAUTI strains.

In this cohort, we hypothesized that some variable genes carried by B2a isolates enhance pathogenic potential in catheterized patients. VF genes previously associated with pathogenic gains of function were identified from a list derived from bacterial pathogenesis literature (28). We identified 32 such VFs in our isolates (Figure 1). The number of VFs per isolate, previously called the “virulence score” (27), did not distinguish (*P* = 0.147) CAUTI (9.5 ± 3.8), CAASB (8.8 ± 3.6), or RC (9.5 ± 4.3) isolates. B2 strains exhibited higher virulence scores than non-B2 strains (10.7 ± 2.7 versus 5.8 ± 4.0, *P* = 0.002), with a nonsignificant trend toward a lower score in B2a than B2b (10.0 ± 2.4 versus 11.5 ± 3.0, *P* = 0.138) (Supplemental Figure 2).

**Figure 2:**
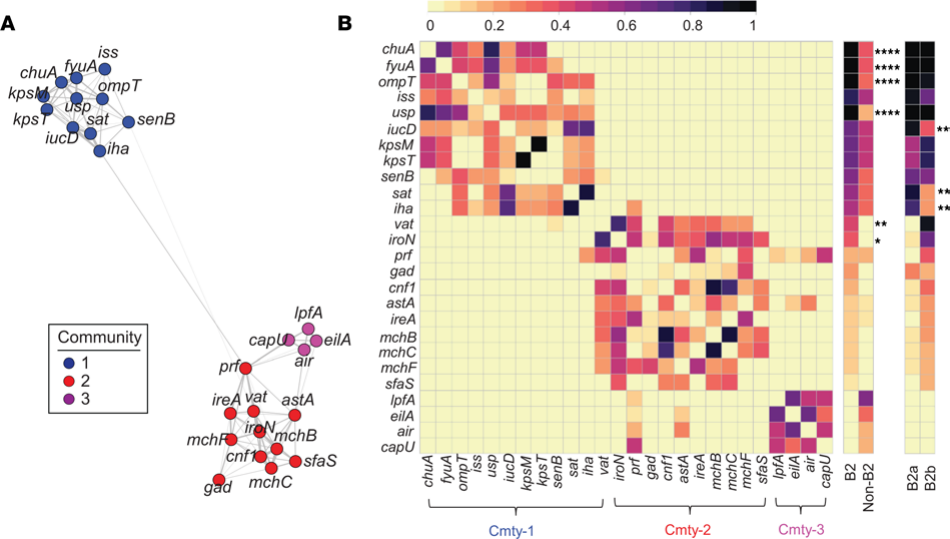
Network analysis of *E*. *coli* VFs. (**A**) A force-directed network layout illustrated coassociations and 3 VF communities among 26 VFs. Each node represented a VF. Each connecting line (edge) represented a positive association between 2 VFs that satisfied the significance threshold (0.4% *P* value threshold, 1-tailed on the right, Fisher’s exact test). Edge lengths were determined by the level of correlation between connected VFs. Nodes were colored by community assignment. (**B**) Three VF communities were discernible in the correlation matrix heatmap depicting statistically significant positive associations between 26 VFs. Presence frequency comparisons of each gene between different genetic groups, B2 versus non-B2 and B2a versus B2b, were displayed in heatmaps to the right of the correlation matrix. Cmty, community. By 2-tailed Fisher’s exact with *P* ≤ 0.05 considered statistically significant. *P ≤ 0.05, **P < 0.01, ****P < 0.0001.

We next considered that VFs may influence pathogenic potential in a nonequivalent manner that includes additive or synergistic VF combinations (28). To assess this, we used network community detection to identify coassociations between the 32 VFs. We used modularity-based community detection using the Louvain method on a weighted network of positive correlations to assess correlations between the 26 VFs that were present more than once (>2.4%) among our isolates (21, 29). Three prominent gene communities were resolved, as visualized by the force-directed network layout (Figure 2A) and its corresponding correlation matrix (Figure 2B). Each community was composed of functionally diverse VFs, with iron acquisition systems and toxins prominent in communities 1 and 2, and with adhesins in all 3. These communities are consistent with a subgroup of VFs that additively or synergistically influence pathogenic potential, though it is also possible that these are lineage markers with no influence on human pathogenicity.

Notably, community 1 and 2 VFs were more common in phylotype B2 strains, while community 3 VFs were exclusively associated with non-B2 strains, with VFs *fyuA*, *chuA*, *ompT*, and *usp* (30–32) being nearly ubiquitous and more common in B2 strains (*P* < 0.00002). In addition, differential relationships between VFs in groups B2a and B2b were evident, with community 1 VFs *iucD,* *sat*, and *iha* being more common in B2a than in B2b (*P* < 0.008) (33–35). These results are consistent with one VF subgroup that increases the pathogenic potential of B2 strains and another VF subgroup that more specifically increases the pathogenic potential of group B2a strains in catheterized patients.

### Biofilm formation by CAUTI, CAASB, and rectal isolates.

**Figure 3:**
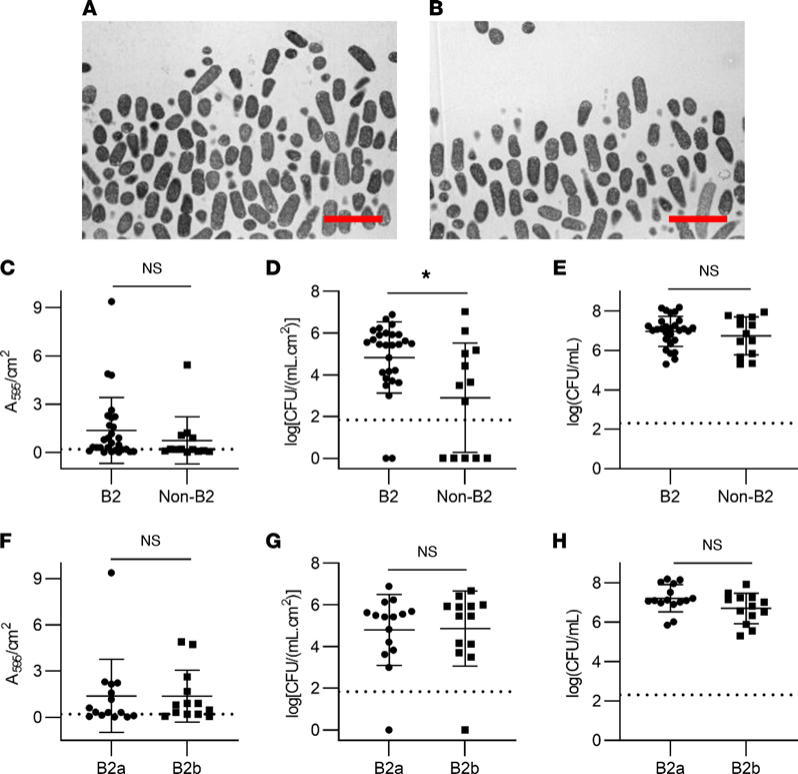
Biofilm formation by CAUTI, CAASB, and rectal isolates. (**A**) Transmission electron microscopy (TEM) image of catheter biofilm grown in human urine. Scale bar: 4 μm. (**B**) TEM image of catheter biofilm grown in artificial urine medium (AUM). Scale bar: 4 μm. (**C**) Comparison of biofilm biomass (crystal violet retention) between B2 and non-B2 isolates. Mean ± SD plotted for 28 B2 and 13 non-B2 strains. *P* = 0.22. (**D**) Comparison of catheter-adherent CFUs between B2 and non-B2 isolates. Mean ± SD plotted for 28 B2 and 13 non-B2 strains. *P* = 0.02. (**E**) Comparison of planktonic CFUs in the voided media between B2 and non-B2 isolates. Mean ± SD plotted for 28 B2 and 13 non-B2 strains. *P* = 0.57. (**F**) Comparison of biofilm biomass (crystal violet retention) between B2a and B2b isolates. Mean ± SD plotted for 15 B2a and 13 B2b strains. *P* = 0.62. (**G**) Comparison of catheter-adherent CFUs between B2a and B2b isolates. Mean ± SD plotted for 15 B2a and 13 B2b strains. *P* = 0.73. (**H**) Comparison of planktonic CFUs in the voided media between B2a and B2b isolates. Mean ± SD plotted for 15 B2a and 13 B2b strains. *P* = 0.13. Statistics were performed with Mann-Whitney *U* test with *P* ≤ 0.05 considered as statistically significant. *P ≤ 0.05.

Catheter biofilm formation is regarded as an important contributor to *E*. *coli* pathogenic potential. *E*. *coli* strains form biofilms that vary in important ways, depending on media and available substrates (16, 18). To evaluate this experimentally, we compared biofilm formation between all 41 *E*. *coli* isolates using an ex vivo, continuous flow model that simulates the clinical catheter environment in patients (Supplemental Figure 3) (36). In this model, we used an artificial urine medium (AUM) (37, 38) that yielded growth kinetics (AUM versus human urine = 7.84 ± 0.19 log[CFU/mL] versus 8.06 ± 0.07 log[CFU/mL], *P* = 0.3, Mann-Whitney *U* test) and biofilm morphology (Figure 3, A and B) comparable with filter-sterilized human urine. Substantial interstrain variation in catheter biofilm formation was evident between isolates, with crystal violet (CV) retention (biofilm biomass) ranging from 0.02 to 10.60 A_595_/cm^2^ and adherent CFUs (sessile bacteria in biofilm matrix) ranging from 0 to 1 ***×*** 10^7.1^ CFU/(mL.cm^2^). Thirty-four of the 41 strains yielded detectable adherent CFUs. Phylotype B2 isolates exhibited significantly higher adherent CFU values (Figure 3D; *P* = 0.02) with a nonsignificant trend (Figure 3C; *P* = 0.22) toward higher CV retention. Neither adherent CFUs nor CV retention values significantly distinguished groups B2a and B2b (Figure 3, F and G; *P* > 0.6), though it is possible that the sample size was insufficient to allow for detection of a difference. Planktonic CFUs (planktonic bacteria in voided media) were not significantly different in all group-wise comparisons (Figure 3, E and H; *P* > 0.1) and were not associated with adherent CFU or CV retention values, possibly reflecting bacterial persistence within loosely adherent communities and/or turbulent flow. These results are consistent with widespread potential for catheter biofilm formation among *E*. *coli* with greater biofilm population size in phylotype B2. Despite the CAUTI and VF gene associations, biofilm formation by B2a strains was indistinguishable from B2b strains.

### CAASB strains can inhibit CAUTI colonization.

The association between CAUTI-associated strains and the subclade B2a genotype, but not biofilm phenotype, may reflect the permissiveness of urinary catheter surfaces for bacterial colonization. In this context, we considered that biofilm-forming isolates with low pathogenic potential from CAASB participants prevent colonization by B2a strains. A protective role for such *E*. *coli* strains is suggested by previous studies (39, 40). This concept has been experimentally tested in patients using *E*. *coli* 83972, a ST73 strain in the B2b subclade (Figure 1) (41) that was isolated from a patient with persistent asymptomatic bacteriuria. *E*. *coli* 83972 has shown efficacy in preventing clinical UTIs following bladder precolonization in patients (42, 43). To determine whether CAASB strains can also prevent colonization by CAUTI strains, we performed a 2-strain competition assay using the continuous flow catheter model. In these experiments, the catheter surface was precolonized by a non-ST131 CAASB biofilm and subsequently challenged with a ST131 CAUTI isolate. We subsequently quantified the ST131 strains in catheter biofilm and planktonic bacterial populations using SNP selective quantitative PCR (SNPs-qPCR) to distinguish them from competing non-ST131 strains.

**Figure 4:**
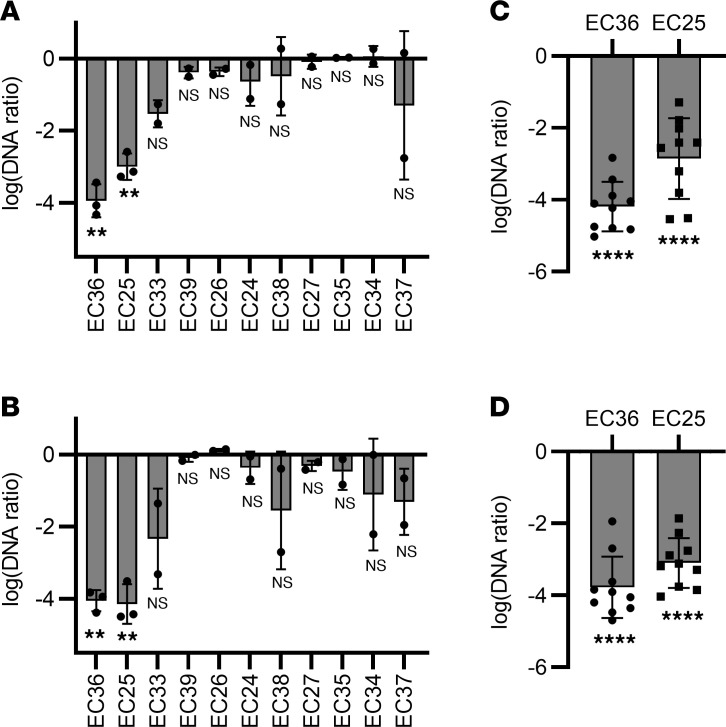
CAASB *E*. *coli* catheter biofilms inhibited CAUTI colonization. (**A** and **B**) DNA ratios of ST131 CAUTI strain EC20 in catheter-adherent bacteria (**A**) and planktonic bacteria (**B**) demonstrate its different levels of colonization when grown by itself (control) and in competition with (competition) 11 non-ST131 CAASB strains (EC36, -25, -33, -39, -26, -24, -38, -27, -35, -34, and -37) in the catheter colonization model. Three replicates with mean ± SD plotted for EC36 and EC25, with *P* < 0.005, 2 replicates with mean ± SD plotted for EC33, -39, -26, -24, -38, -27, -35, -34, and -37, with *P* > 0.10. (**C** and **D**) DNA ratios of 10 ST131 CAUTI strains (EC12, -13, -14, -15, -16, -17, -18, -19, -20, and -22) in catheter-adherent bacteria (**C**) and planktonic bacteria (**D**) demonstrate their different levels of colonization when grown by itself (control) and in competition with (competition) 2 non-ST131 CAASB strains (EC36 and EC25) in the catheter colonization model. Ten ST131 CAUTI strains with mean ± SD plotted, with *P* < 0.0001. DNA ratio = (DNA_competition_)/(DNA_control_). Statistics were performed with 1-sample *t* test. *P* ≤ 0.05 is considered statistically significant. **P < 0.01, ****P < 0.0001.

We first assessed the ability of 11 non-ST131, non-B2a strain CAASB isolates to inhibit catheter colonization by ST131 CAUTI isolate EC20, a high-biofilm former. Total CFUs, representing both strains, were similar between different pairwise bacterial competition cultures for both catheter and planktonic populations (Supplemental Figure 4). Precolonization with EC36 (phylotype B2) and EC25 (phylotype A) significantly inhibited both catheter-adherent (Figure 4A; *P* < 0.005) and planktonic EC20 populations, as assessed by SNPs-qPCR (Figure 4B; *P* < 0.006). Precolonization with EC36 or EC25 also suppressed both catheter biofilm (Figure 4C; *P* < 0.0001) and planktonic representation (Figure 4D; *P* < 0.0001) of all 10 CAUTI ST131 isolates. These results are consistent with the ability of a subset of CAASB strains (2 of 11; 18%) to markedly prevent catheter colonization and shedding by ST131 CAUTI strains with antibiotic resistance and elevated pathogenic potential (41). This raises the possibility that *E*. *coli* catheter biofilm formation in asymptomatic patients may play a protective role by preventing colonization with *E*. *coli* of greater pathogenic potential.

### Identification of catheter biofilm–associated genes.

**Figure 5:**
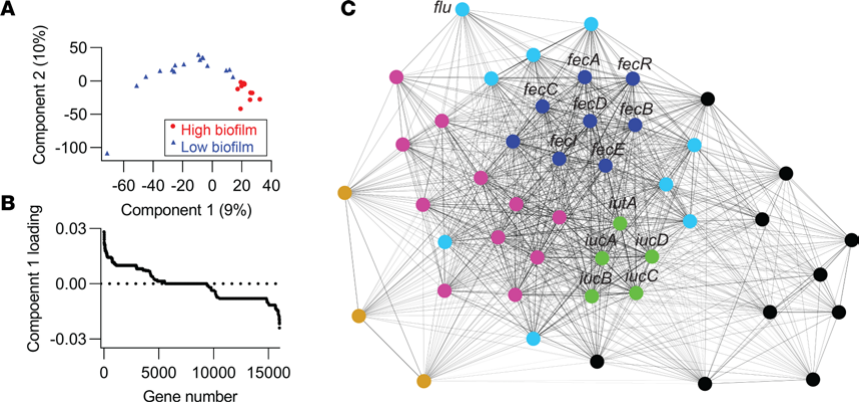
Identification of catheter biofilm–associated genes. (**A**) Score plot of the first 2 components from sparse partial least squares discriminant analysis (sPLSDA) for displaying group-wise clustering between high- and low-biofilm formers. (**B**) Component 1–associated top loadings from sPLSDA identified 72 biofilm-correlated genes, including 46 positive (high-biofilm) and 26 negative (low-biofilm) genes. (**C**) A force-directed network layout illustrated coassociations and 3 gene communities among 46 biofilm positively associated genes. Each node represented a gene. Each connecting line (edge) represented a positive association between 2 genes that satisfied the significance threshold (5% *P* value threshold, 1-tailed on the right, Fisher’s exact test). Edge lengths were determined by the level of correlation between connected genes. Nodes were colored by community assignment.

Genomic and phenotypic results suggest that catheter biofilm formation may play a role in both the promotion and prevention of CAUTI, depending upon the presence of specific virulence gene combinations. To determine whether there also exists a distinctive set of catheter biofilm–associated genes, we conducted a comparative genomic analysis of strains with high or low catheter biofilm formation. We selected isolates with high- and low-biofilm formation from each of the 5 main clades (Figure 1) — B2a, B2b, F, D, and A + B1 — based on their CV retention (biofilm biomass) values (Table 3). The criteria of “CV < 0.2” and “CV > 1” were adopted for low- and high-biofilm formers, and they identified 13 high- and 16 low-biofilm isolates, respectively (Table 3). We next compared genome composition between these 2 groups using sparse partial least squares discriminant analysis (sPLSDA) (44). In the sPLSDA score plot, high- and low-biofilm formers were well resolved along the PC1 axis (Figure 5A). Classification between high- and low-biofilm formers by LR using PC1 (9%; Supplemental Figure 5A) values yielded a prediction accuracy of 1.0 (Supplemental Figure 5B; SD = 0) and an AUC of 1.0 (Supplemental Figure 5C; SD = 0) with 5-fold cross validation. Seventy-two genes with varied functional associations and significant PC1 loadings (*P* < 0.05 by 2-tailed Fisher’s exact test) were detected, with 46 and 26 genes associated with positive (high biofilm) and negative (low biofilm) PC1 loadings, respectively (Figure 5B and Supplemental Table 4). Of note, the antigen 43 gene (*flu*) (45) was among the positively associated genes, with the 44th highest PC1 loading (*P* = 0.04 by 2-tailed Fisher’s exact test), providing a confirmatory point of reference to a previously described *E*. *coli* biofilm–associated gene.

**Figure 6:**
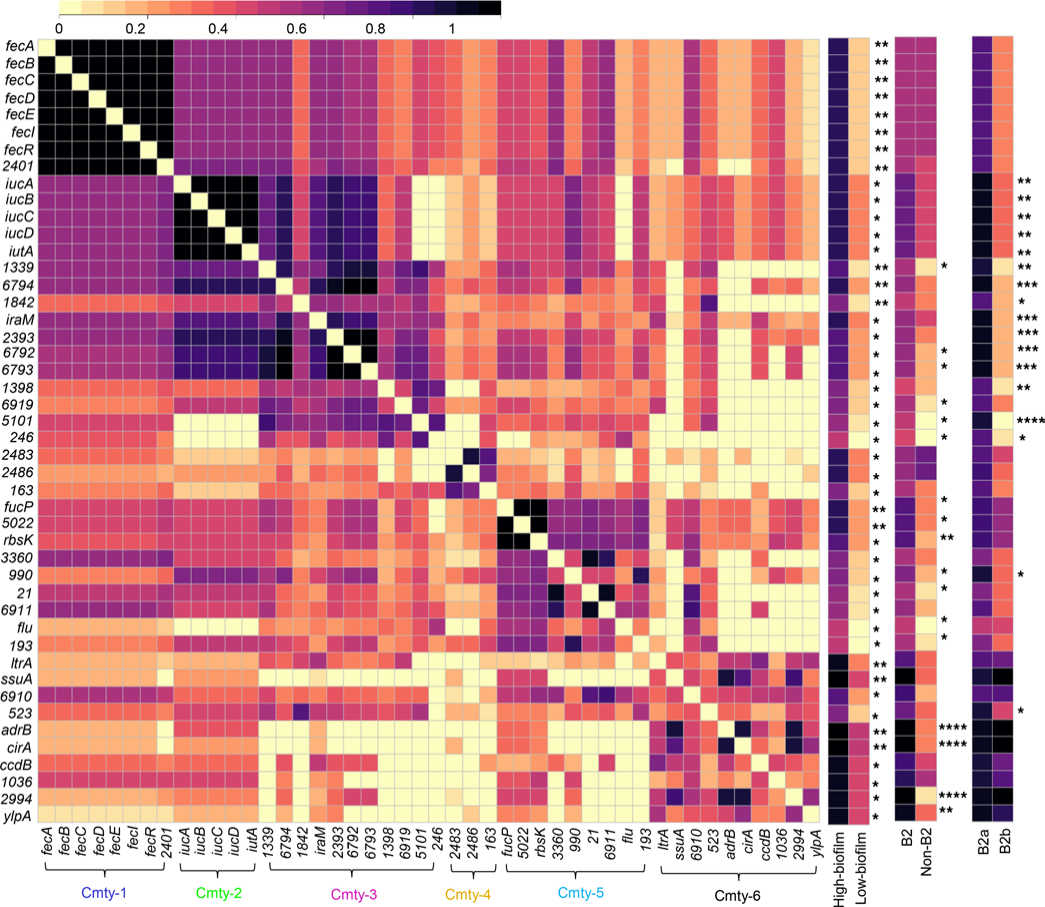
Six gene communities are discernible in the correlation matrix heatmap of catheter biofilm–associated genes. The correlation matrix heatmap depicts statistically significant (5% *P* value threshold, 1-tailed on the right, Fisher’s exact test) positive associations between 46 biofilm positively associated genes. Presence frequency comparisons of each gene between different phenotypic and genetic groups, high-biofilm versus low-biofilm, B2 versus non-B2, and B2a versus B2b, were displayed to the right of the correlation matrix. Cmty, community. Statistics were performed with 2-tailed Fisher’s exact, with *P* ≤ 0.05 considered statistically significant. *P ≤ 0.05, **P < 0.01, ***P < 0.001, ****P < 0.0001.

To identify coassociations between the 46 genes associated with high catheter biofilms, we performed modularity-based community detection using the Louvain method on a weighted network of positive correlations, as described above for VFs (21, 29). This analysis resolved 6 gene communities, visualized by the force-directed network layout (Figure 5C) and its corresponding correlation matrix (Figure 6). Community 1 was composed of the ferric citrate transport locus (*fecABCDIR*) (46, 47) and exhibited robust coassociations and the highest betweenness centrality ranking in the weighted network. The *fec* genes also exhibited the strongest association with high catheter biofilm formation in the sPLSDA analysis (*P* = 0.0025), suggesting a major role in this biofilm phenotype. Community 2 defined the aerobactin siderophore system locus (33) represented by the VF marker gene *iucD*. Relative to communities 1 and 2, communities 3–6 were less robust and are composed of genes associated with more divergent functions.

Together, these results are consistent with a complex polygenic contribution to catheter biofilm production that features a prominent role for iron transport systems (ferric citrate and aerobactin systems, *cir*) and autoaggregation (antigen 43/*flu*) (Supplemental Table 4). The significant positive associations between multiple biofilm genes, B2, and B2a strains (Figure 6) suggests that biofilm-associated genes may play a contributing role in the pathogenic potential of CAUTI isolates.

### Fec system activity is associated with increased catheter biofilm formation.

**Figure 7:**
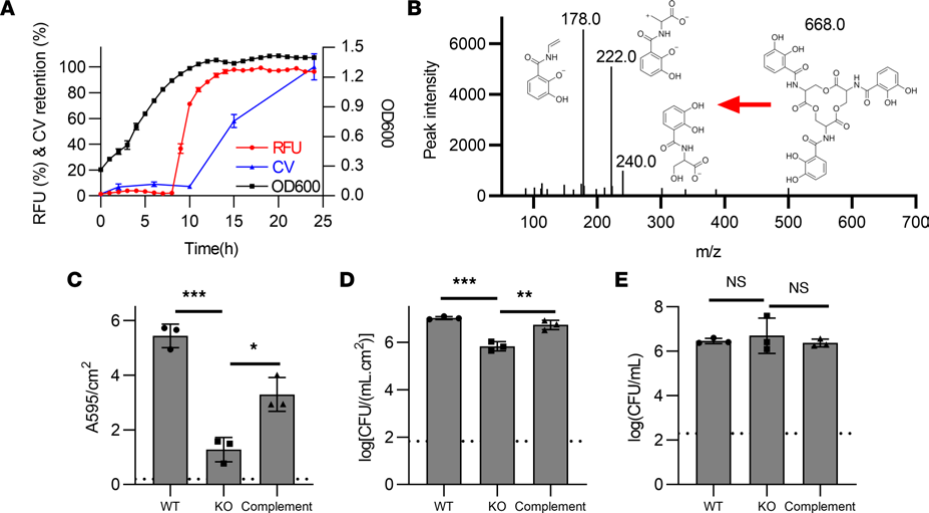
Fec expression and extent of catheter biofilm formation. (**A**) The *fec* expression (RFU), catheter biofilm formation (CV retention, A595/cm^2^), and bacterial growth (OD600) by the fluorescent reporter strain EC52:*fecI*-RFP was measured in a microplate assay. (**B**) MS/MS product ion scan spectrum demonstrating the presence of the *E*. *coli* siderophore enterobactin (*m/z* = –668.1) in the voided media from WT EC52 cultured in the continuous flow catheter biofilm assay system. (**C**–**E**) Biofilm biomass (crystal violet retention, **C**), catheter-adherent CFUs (**D**), and planktonic CFUs (**E**) of EC52 (WT), EC52Δ*fecA* (KO), and EC52Δ*fecA*:*fecA* (complement) cultured in the catheter biofilm assay system. Three replicates with mean ± SD plotted. Comparisons conducted using 1-way ANOVA with Dunnett’s multiple-comparison test. *P* ≤ 0.05 is considered statistically significant. *P ≤ 0.05, **P < 0.01, ***P < 0.001.

The positive association between catheter biofilm and *fec* genes raises the possibility that the Fec system plays a causative role in enhancing catheter biofilm formation (46, 47). To address this, we examined the Fec expression profile in a WT strain and measured catheter biofilm formation in a *fecA*-deficient mutant. The Fec system imports extracellular ferric citrate complexes to the periplasm through the outer membrane transporter FecA, after which ferric citrate activates the sigma factor FecI through the inner membrane protein FecR, followed by transcriptional activation of *fecABCDE*, which facilitates cytoplasmic iron delivery (46–48). To determine whether *fec* gene transcription is associated with *E*. *coli* biofilm formation, we constructed a FecI-dependent fluorescence reporter strain (EC52:*fecI-RFP*), in the *fec*^+^ high-biofilm rectal isolate, EC52. Shaking microplate cultures of EC52:*fecI-RFP* exhibited abrupt fluorescence activation upon stationary phase entry at 8 hours, immediately preceding detectable biofilm formation (Figure 7A). These observations temporally connect transcriptional activation of *fec* genes to *E*. *coli* biofilm formation.

To determine whether Fec affects catheter biofilm formation, we next sought to determine whether iron acquisition systems are active in the biofilm culture system. We assessed this by determining whether enterobactin, the conserved *E*. *coli* siderophore excreted under low iron conditions, is secreted by bacteria during catheter biofilm culture. Using liquid chromatography–mass spectrometry (LC-MS), we detected established MS/MS ions for enterobactin (49, 50) in voided media from catheter biofilm produced by EC52, EC52Δ*fecA*, and complemented EC52Δ*fecA* at 10 hours of growth, consistent with activation of *E*. *coli* iron acquisition systems (Figure 7B and Supplemental Figure 6). To determine whether the Fec system influences catheter biofilm formation, we next compared biofilm formation by isolate EC52 to its isogenic *fecA* deletion mutant EC52Δ*fecA* in the catheter continuous flow system (47). Both CV retention (Figure 7C; *P* = 0.0001) and sessile bacterial counts (Figure 7D; *P* = 0.0002) were significantly lower in EC52Δ*fecA* relative to WT EC52. Genetic complementation of EC52Δ*fecA* with a *fecA* expression plasmid (EC52Δ*fecA:fecA*) significantly reversed this biofilm formation deficit (Figure 7, C and D; *P* < 0.006). No significant differences in planktonic CFUs (nonbiofilm growth) were observed between WT, mutant, and complemented strains (Figure 7E; *P* = 0.7), consistent with indistinguishable growth curves in AUM for these 3 strains (Supplemental Figure 7). These data are consistent with activation of iron uptake systems during catheter biofilm formation and suggest a role for the Fec system in catheter biofilm formation.

## Discussion

In this study, we identified a genomic lineage within the *E*. *coli* B2 phylotype that is associated with CAUTI in a hospitalized population. This lineage is dominated by pandemic, multidrug-resistant ST131 strains (24, 25) that possess a distinctive combination of VF genes. We found that experimental catheter biofilm formation (35) did not distinguish ST131 strains and that biofilms produced by a subset of non-ST131 CAASB strains could prevent colonization by CAUTI-associated ST131 strains. In comparative metagenomic analyses, we found biofilm-associated genes to be largely distinct from those associated with CAUTI, consistent with a possible shared role for biofilm in both CAASB and CAUTI. Notably, different iron-responsive gene systems were associated with both CAUTI and biofilm formation. The ferric citrate transport system, Fec, was the most prominent catheter biofilm correlate, was transcriptionally activated early in biofilm formation, and was functionally associated with enhanced catheter biofilm formation. The overall results are consistent with a multifaceted role for *E*. *coli* biofilm formation in colonizing catheterized hosts, with an elevated risk for infectious progression by ST131 strains carrying a distinctive combination of virulence-associated genes. In this paradigm, catheter biofilm formation by *E*. *coli* may be protective in some patients and harmful in others, depending upon the presence of specific virulence function combinations.

Identification of a distinct, infection-associated *E*. *coli* lineage in a clinical *E*. *coli* bacteria cohort at this degree of resolution is unusual (20, 27). This result may reflect the study’s singular focus on catheterized patients, in whom infection may arise through a relatively distinct and uniform pathophysiology in a more homogenous host population. The abundance of ST131 strains in this study may also reflect their relatively recent global proliferation (25), aided by an ability to efficiently colonize and persist in human intestinal reservoirs (51–54), which is regarded as the source of most urinary *Enterobacterales* (55–57). While ST131 intestinal colonization exhibits no sex differences (58–60), CAUTI patients in this study were disproportionately male, possibly reflecting the enhanced *E*. *coli* infection severity in males observed in an animal model of direct bladder inoculation (61, 62). It is unclear whether the association between ST131 strains and CAUTI arises from increased pathogenic potential of these strains, an association with male patients, or a combination thereof. CAUTI-associated ST131 strains compared with CAASB strains are not distinguished by their ability to form catheter biofilms in the present study but, rather, by a combination of accessory genes, including VFs, suggesting a potential role for enhanced virulence. Because blood cultures are seldom obtained from asymptomatic individuals, a sufficiently powered study to more closely distinguish these possibilities in male CAASB and CAUTI patients would likely require obtaining prospective urine cultures from asymptomatic patients.

Of note, the ability of some strains to interfere with ST131 catheter colonization raises the possibility that catheter biofilms formed by *E*. *coli* strains without high-risk virulence gene combinations may benefit patients. Although 1 such strain that has been extensively studied in this regard, *E*. *coli* 83972 (42, 43), was collected from a noncatheterized patient with 3 years of bacteriuria, the current study suggests that protective strains are common in catheterized patients. High antibiotic resistance among ST131 strains (63) raises the possibility that antibiotic treatment preferentially eliminates protective *E*. *coli* strains while sparing ST131 strains, paradoxically increasing the likelihood of progression to CAUTI. This scenario further reinforces guideline recommendations to be judicious with antibiotic use (1, 4, 7). A clinical test distinguishing ST131 from non-ST131 bacteriuria could also aid treatment decisions by helping to differentiate CAUTI and CAASB, a stated area of diagnostic need (63).

The processes that predispose biofilm-bound *E*. *coli* to progress to CAUTI remain unclear but are of diagnostic and therapeutic interest (15). These processes are presumably complex and include biofilm efflux, host tissue adhesion, immune evasion, and nutrient acquisition (64). VFs in this study did not appear to equip strains for infection as equally influential components with simple additive effects on pathogenic potential. Neither the number of VFs nor their general functional categories clearly distinguish B2a from B2b strains. B2a and B2b strains are, however, distinguished by the presence of specific VF combinations encoding siderophore, adhesin, and toxin systems. If VFs affect pathogenic potential in catheterized patients, this appears to occur through idiosyncratic functions of specific VFs acting within evolutionarily favored combinations, suggested by the presence of VFs in the favored network communities described here (Figure 2) and in previous work (21, 29). Previously identified VFs are not the only possible contributors to pathogenic potential in ST131 strains. B2a strains in this study carry 224 unique genes that are absent in B2b strains that may also modulate pathogenic potential. Discerning the contributions of these genes would require further study.

The variable genes associated with biofilm formation are substantially different from those associated with CAUTI. In the present study, the Fec was the most prominent of these, with 2 other iron acquisition systems, the aerobactin siderophore system and the ferric catecholate importer Cir (65), also represented. The deficiency in catheter biofilm formation by a Fec-deficient mutant that produces enterobactin, the prototypical *E*. *coli* siderophore, was surprising, since previous work indicates that this deficiency was only discerned in planktonic, siderophore-deficient *E*. *coli* mutants (66, 67). These discrepant observations may relate to important differences in iron acquisition and trafficking in the biofilm matrix. Consistent with our observation here, a recent study identified *fecA* as an *E*. *coli* fitness factor in a murine UTI model despite retained enterobactin function (68). Precisely how these different iron acquisition–related systems function in the context of a catheter biofilm remains unclear. It is possible that, in biofilm microenvironments, the lower metabolic cost of citrate as an iron chelator is an important feature and that host-derived urinary citrate favors bacteria that are able to use this “free” resource (46, 47). It is also possible that the Fec system mediates biofilm-specific functions independently of its ability to mediate iron uptake. Together, investigating the Fec as well as other iron acquisition systems in UPEC provide great insights for better elucidating bacterial pathogenesis in UTI and CAUTI, aiding in the search for new therapeutic approaches.

In conclusion, we found that CAUTI in the study population was associated with *E*. *coli* lineage largely defined by emergent multidrug-resistant ST131 strains. The pathogenic potential of these populations was associated with carriage of specific gene networks and a high degree of fluoroquinolone resistance, an antibiotic class commonly used to treat UTIs. ST131 strains appeared well adapted to cause infection in patients with urinary catheters, raising the possibility that these strains arose from coevolution with catheterized human hosts. The gene networks associated with biofilm formation were largely distinct from the CAUTI-associated gene networks. In addition, catheter biofilm formation was widespread among *E*. *coli* strains, and some strains in asymptomatic bacteriuria could act to prevent the colonization by CAUTI-associated ST131 strains. These results suggest that strain-specific characteristics of urinary *E*. *coli* influence CAUTI pathogenesis in patients. Strain-specific testing may, thus, aid clinical decision making in this population. An improved understanding of how ST131 strains cause infections may suggest future therapeutic strategies for these increasingly antibiotic-resistant bacteria.

## Methods

### Urinary isolates.

Urinary catheter-associated *E*. *coli* isolates were identified from a previously described study of bacteriuric (> 5 ***×*** 10^4^ CFU/mL) inpatients (5, 6). CAUTI was defined as fever (T > 38°C) with contemporaneous bacteriuria and urinary catheter placement (69). Documented urinary symptoms (dysuria, lower abdominal pain, flank pain) in the absence of fever were regarded as insufficient for CAUTI diagnosis due to their poor reliability in inpatients, particularly those with urinary catheters (1, 4, 7). CAASB was defined as bacteriuria in the absence of fever and documented urinary symptoms.

### Rectal isolates.

Rectal *E*. *coli* isolates were collected from healthy adult volunteers in St. Louis, Missouri, USA, from 2014 to 2015. Exclusion criteria included age < 18 years old, pregnancy, current urinary tract infection, previous urogenital surgery, ongoing treatment for urogenital cancer, the use of systemic antibiotics within 30 days of the study visit, or the use of a urinary catheter within 30 days of the study visit. Each study participant used a previously published protocol (70) to procure a self-collected rectal swab (BD Eswab) and submitted it with a study survey. Swabs were processed by the clinical microbiology lab at Barnes-Jewish Hospital to identify a dominant *E*. *coli* isolate and assess its antibiotic susceptibilities. Fifty-seven individuals were consented, 48 individuals submitted study materials, 41 *E*. *coli* isolates had matching demographic data, and 13 of those *E*. *coli* isolates were randomly selected for the current study.

### Human urine.

Healthy donor urine was collected from adult volunteers, as approved by the WU-IRB. Participants provided written informed consent for collection of up to 2 specimens, at least 1 week apart, for subsequent discovery and validation analysis. Exclusion criteria included recent UTI, antibiotic therapy, pregnancy, or any urogenital diseases. Collected human urine were mixed together, filter sterilized (MilliporeSigma Steritop Filter, 0.22 μm), and stored in –80°C until use. Before use in an experiment, frozen urine specimens were thawed on ice and filter sterilized again (71).

### Bacterial strains and culture.

An isogenic mutant of *E*. *coli* strain EC52 was constructed as in-frame deletion using the lambda red recombinase method, as described previously (72). Isogenic mutant complementation and fluorescence reporter construct were accomplished by ectopic expression using transformed plasmids (47). Unless otherwise specified, cultures were grown from single colonies in LB broth for 12 hours at 37°C before using in the indicated assays.

### Whole-genome sequencing.

Bacterial genomic DNA was extracted with a QIAmp BiOstic Bacteremia DNA kit (Qiagen) from ~10 colonies of overnight growth. In total, 5 ng of DNA was used as input to create Illumina sequencing libraries using the Nextera kit (Illumina). The samples were pooled and sequenced on an Illumina NextSeq 500 High Output system to obtain 2 ***×*** 150 bp reads. The reads were demultiplexed by barcode and had adapter sequences removed with trimmomatic v.38 and contaminating sequenced removed with deconseq v.4.3 (73). Processed reads were assembled into draft genomes with SPAdes v3.12.0 (Bankevich). The scaffolds.fasta file from spades was annotated for protein coding sequences on all contigs > 500 bp with prokka v1.12 (74). Additionally, we obtained *E*. *coli* genomes in the known phylogroups and annotated their protein coding sequences. GFF files from prokka were used as input for roary to create a core-genome alignment with PRANK (75). The core-genome alignment was constructed into a maximum likelihood tree with raxML and viewed in iTOL (76). In silico multilocus STs (MLST) were identified using BLASTN to the *E*. *coli* MLST database (23). Previously published VFs were annotated in the *E*. *coli* draft genomes using virulencefinder v1.5 and blastp to previously described genes (77). ARGs in genomic assemblies were identified by BLAST comparison of protein sequences against the CARD database based on stringent cutoffs (> 95% identity and > 95% overlap with subject sequence) (78).

To examine clonality among B2a subclade isolates, Roary was repeated on the GFF files of the 15 B2a isolates to produce a core-genome alignment of 3,588 genes. SNP-Sites (https://github.com/sanger-pathogens/snp-sites; branch name, master; commit ID, 52c98cb) was run on the alignment file to produce a VCF that identified 3,351 total SNPs within this cohort (79). Pairwise SNP distances were calculated for all genomes using vcfR (80) and custom Python scripts as described in D’Souza et al (81). The genomes analyzed in this report have been deposited to NCBI WGS database under BioProject accession no. PRJNA514354.

### Genomic analysis.

The 32 VFs were compared between phenotypic and genetic groups for identifying CAUTI-associated VFs using sPCA, LR classification, and network analysis approaches. Biofilm-associated genes were determined by comparative genomic analyses using sPLSDA, LR classification, and network analysis approaches (21, 26, 27, 30). Computational models used in these genomic analyses were configured in Python and R programming languages, mainly by using the scikit-learn module and mixOmics packages, respectively, as well as the Gephi software (http://gephi.org). Because of the high sparsity of genomic metadata, with 15,993 genes identified in 41 genome assemblies (15,993 >> 41), sparse penalty was enforced in all dimensionality reduction analyses (sPCA and sPLSDA) to prevent overfitting (82).

### Network analysis.

Two network representations for the 26 VFs and 46 high-biofilm genes, connected by cooccurrences across the *E*. *coli* collection, were defined using statistically significant positive correlations as the edge weights across the networks. Statistical significance between 2 nodes (genes) was determined by Fisher’s exact test to determine whether they appeared independently, conditional on their observed marginal frequencies among the *E*. *coli* collection. The 0.4% and 5% *P* value thresholds (1-tailed on the right) were chosen for the VFs and biofilm^+^ genes networks, respectively, to ensure that the obtained gene network in each case was a single connected component. An edge was defined as present between any pair of positively correlated nodes that satisfied the significance threshold, with edge weight equal to the positive correlation coefficient. Communities in this network were detected using the Louvain method by maximizing the modularity function (21, 29). We selected the obtained 3-community (Resolution = 1.0) and 6-community (Resolution = 1.25) for the network visualizations of 26 VFs and 46 high-biofilm associated genes, respectively, using a force-directed layout generated by the Gephi (http://gephi.org) ForceAtlas2 algorithm and the corresponding correlation matrix.

### AUM.

AUM (Supplemental Table 5) was prepared as an alternative medium of human urine for characterizing biofilm formation. For our AUM formulation, we adjusted the iron and zinc content of a previously published AUM (37, 38) to more closely approximate that of human urine specimens (83). The earlier (37) AUM formulation contains more iron and less zinc than pooled human urine (83) (iron is 5 µM versus 0.21 µM; zinc is 0 µM versus 7.0 µM). By omitting ferric salt supplementation and adding 7 μmol/L ZnSO_4_.7H_2_O (Supplemental Table 5), we produced an AUM with iron and zinc concentrations of 0.86 ± 0.14 μM and 9.60 ± 0.89 μM, which are closer to physiological values. ICP-MS experiments were conducted at the Nano Research Facility (NRF), Department of Energy, Environmental and Chemical Engineering, Washington University in St. Louis. ICP-MS quantification was achieved using calibration curves of 1, 5, 10, 50, and 100 μg/L. Nitric acid (Thermo Fisher Scientific) was added into pooled human urine samples with a final acid concentration of 2% (71)

### Continuous-flow catheter biofilm model system.

Biofilms were grown in a continuous-flow catheter model, using previously published protocols with appropriate modifications (Supplemental Figure 3) (36). The components used for assembling the continuous-flow system included the platinum-cured silicone urinary catheters (Nalgene 50), peristaltic pump (Watson-Marlow, 205U), flexible tubings (Tygon S3), and plastic connectors (Thermo Fisher Scientific). Prior to use, all tubing, connectors, and containers were autoclaved. Human urine and AUM were filter sterilized (MilliporeSigma Steritop Filter, 0.22 μm). Bacteria from single colonies were grown in LB broth under 37°C for 12 hours, washed with PBS, back diluted 1:10 into filter-sterilized human urine or AUM, and injected into the catheter installed in the continuous-flow system operating under 37°C. *E*. *coli* inoculum was statically incubated for 2 hours to allow the bacterial attachment to catheter surface. Fresh medium was then pumped through the catheter at the flow rate of 0.5 mL/min, with a 30-minute preflush to first wash off loosely adherent bacteria. After 10 hours of continuous flow incubation, voided media and catheters were collected for characterization.

Total biofilm biomass was quantified by CV retention. Biofilm-bound (sessile) and unbound (planktonic) bacterial counts were determined by CFU enumeration of the biofilm matrix or voided media (36). Catheter (11 cm) collected from the flow system was washed with PBS and cut into 3 pieces (3 cm), with 2 pieces for CV and 1 for CFU assays. For CV staining, 3 cm catheters were stained with 0.5% CV solution for 10 minutes, washed with deionized water, air-dried on absorbent paper overnight, and extracted with 33% acetic acid for 10 minutes. CV extracts were diluted 20-fold and measured at 595 nm using a Spectrophotometer (Beckman Coulter, DU-800). To quantify sessile bacteria, 3 cm catheter was cut into fragmented pieces, immersed in 3 mL PBS, sonicated (Branson 350) for 10 minutes, vortexed for 3 minutes (GeneMate), and plated to quantify as CFU/(mL × cm^2^). To quantify planktonic bacteria, voided media were collected and directly plated for CFU enumeration as CFU/mL.

### Biofilm structure characterization.

Biofilm grown on urinary catheter surface was processed for structure characterization using transmission electron microscopy (TEM) (84). For microstructural characterization, 1 cm catheter with biofilm formed on the inside surface was fixed in 2% paraformaldehyde/2.5% glutaraldehyde (Polysciences Inc.) in 100 mM sodium cacodylate buffer (pH 7.2) for 1 hour at room temperature. Samples were washed in sodium cacodylate buffer and postfixed in 1% osmium tetroxide (Polysciences Inc.) for 1 hour. Samples were then rinsed extensively in deionized water prior to en bloc staining with 1% aqueous uranyl acetate (Ted Pella Inc.) for 1 hour. Following several rinses in deionized water, samples were dehydrated in a graded series of ethanol and embedded in Eponate 12 resin (Ted Pella Inc.). Sections of 95 nm were cut with a Leica Ultracut UCT ultramicrotome (Leica Microsystems Inc.), stained with uranyl acetate and lead citrate, and viewed on a JEOL 1200 EX transmission electron microscope (JEOL USA Inc.) equipped with an AMT 8 megapixel digital camera and AMT Image Capture Engine V602 software (Advanced Microscopy Techniques).

### Bacterial interference analysis.

Competitive colonization between 11 non–ST131 CAASB and 10 ST131 CAUTI isolates (Supplemental Table 6) was evaluated in the continuous flow catheter biofilm system (Supplemental Figure 3B) with 2 consecutive flow stages (Supplemental Figure 8) (85). All flow experiments were conducted aseptically under 37°C using AUM. Urinary catheters were precolonized with a CAASB biofilm for 10 hours and were then challenged with a CAUTI isolate for another 10 hours. At this endpoint, voided media and urinary catheters were collected to determine total CFU and the proportion of CAASB and CAUTI strains by SNPs-qPCR as described below. The proportions of CAASB and CAUTI bacteria in both planktonic and biofilm 2-bacteria pellets were used to measure the affect of precolonized CAASB strain biofilm on CAUTI strain colonization.

### SNPs-qPCR.

We quantified CAASB and CAUTI strain proportions in mixed cultures using SNPs-qPCR. Housekeeping genes (25) were aligned to identify SNP-containing segments to distinguish between 2 strains. Two housekeeping genes *adk* (adenylate kinase) and *gyrB* (DNA gyrase) were identified with strain-specific SNPs that could differentiate the 11 non-ST131 CAASB from the 10 ST131 CAUTI isolates (Supplemental Figure 9). Three SNP-containing portions in *adk* distinguished EC24, EC25, EC26, EC27, EC37, EC38, and EC39 from the 10 CAUTI isolates. Two SNP-containing portions in gene *gyrB* differentiated EC33, EC34, EC35, and EC36 from the 10 CAUTI isolates (Supplemental Table 7). Primers (Supplemental Table 8) amplifying the SNP-containing portion in each gene were designed and validated by PCR following gel electrophoresis to confirm that SNPs-qPCR assays distinguish between 2 *E*. *coli* isolates in mixed cultures (Supplemental Figure 10).

Prior to qPCR, bacteria cultures collected from catheter biofilm system were spun down to collect 2-bacteria pellets, and DNA was extracted using Wizard Genomic DNA Purification Kit (Promega) and measured by NanoDrop 2000 Spectrophotometer (Thermo Fisher Scientific). All qPCR assays were performed on a CFX96 Real-Time System (Bio-Rad). The 20 μL PCR mixture contained 1× iTaq Universal SYBR Green Supermix (Bio-Rad), 0.2 μM of each primer, and 3 ng/μL DNA of each specimen. The standard running conditions consist of a 3-minute polymerase activation and DNA denaturation at 95°C, another 10-second DNA denaturation at 95°C, 40 cycles of a 30-second annealing at 58.5°C, ending with a melt curve with 5 seconds at 65°C first and 5 seconds each at a 0.5°C increase between 65°C and 95°C (86), with threshold cycles (Cq) obtained at the end of the reactions. Calibration curves (log[Cq] ~ log[DNA]) for each strain’s SNPs-qPCR assay were established at 0.09375, 0.1875, 0.75, 1.5, and 3 ng/μL (Supplemental Table 7). The acquired linear calibration curves demonstrated the ability of each SNPs-qPCR assay to detect the expected proportions of bacteria in mixed cultures. Finally, in the mixed cultures of 2 *E*. *coli* isolates, Cq were obtained to determine the quantity of DNA (ng/μL) for each isolate, with quantification using the calibration curve.

### The fec fluorescence and microplate-biofilm assays.

Fluorescence from the *fecI* red fluorescent protein (RFP, mCherry) reporter was measured to assess transcriptional activation of the *fec* pathway (47). The *fec* reporter plasmid was transformed into isolate EC52, a high-biofilm former, to create the reporter strain, EC52:*fecI*-RFP (Supplemental Table 9). A control strain, EC52:RFP, was constructed using the same plasmid without the *fec* promoter managing RFP expression (Supplemental Table 9). Primers (Supplemental Table 8) used to construct the plasmids were designed and validated by PCR following gel electrophoresis. These strains were cultured in a Tecan Spark microplate reader at 37°C to monitor *fec* expression levels at different stages of bacterial growth in M63/0.2% glycerol minimal medium over 24 hours. To relate *fec* expression to biofilm formation, CV determinations were performed as described above over a time series.

### Mass spectrometry.

Eluates from the catheter biofilm system were promptly centrifuged to remove bacteria and particulates (21,000*g* for 2 minutes at 4°C), filtered (0.45 μm Millex PVDF Durapore syringe-driven filter), and stored at –80°C prior to analysis. Samples were analyzed by LC-MS with a Thermo Vanquish ultrahigh pressure liquid chromatograph interfaced with a Thermo ID-X Tribrid mass spectrometer with an ESI source (87). Chromatography was performed using a Ascentis-Express fused core phenyl-hexyl column (100 mm ***×*** 2 mm ***×*** 2.7 μm) with a 0.5 mL/min flow rate. The column was equilibrated in 95% A (0.1% [v/v] formic acid) and 5% B (90% acetonitrile plus 0.1% [v/v] formic acid) prior to sample injection. Percent buffer B was held at 5% until 2 minutes; it then increased to 56% at 10 minutes and 98% at 12 minutes. The column was held at 98% B until 16 minutes, and it then returned to 5% B by 18 minutes and held until 21 minutes. Negative ion MS/MS were collected for the precursor ion –668.1 *m/z* (cyclic enterobactin). Product ions were extracted and integrated using Thermo TraceFinder software version 5.1.

### The fec deletion mutants.

*fecA*, encoding the ferric citrate outer membrane receptor, was deleted from the high-biofilm–forming strain EC52 using Lambda Red recombinase method, creating the isogenic mutant EC52Δ*fecA* (Supplemental Table 8) (72). EC*52*Δ*fecA* was genetically complemented using a *fecA* expression plasmid, generating EC52Δ*fecA*:*fecA* (Supplemental Table 8) as a control. Primers (Supplemental Table 9) used in the *fecA* deletion and complementation were designed validated by PCR and gel electrophoresis.

### Data availability.

The genomes analyzed in this report have been deposited to NCBI WGS database under BioProject accession no. PRJNA514354. The computer codes for the analyses in this study are available in Github (https://github.com/QL5001/CAUTI-script; branch name, main; commit ID, c673712).

### Statistics.

GraphPad Prism 8.0 (GraphPad software) was used to generate graphs and perform statistical analysis in this study. We used 1-sample *t* test for single group comparison, Mann-Whitney *U* test for 2-group comparisons, and 1-way ANOVA for multigroup comparisons. Dunnett’s tests was used to correct 1-way ANOVA multigroup comparisons where appropriate. *P* < 0.05 was considered significant.

### Study approval.

Patient bacterial specimens were collected with the approval of the Washington University IRB. Study design, inclusion and exclusion criteria for urinary isolates has been previously described (5, 6). All participants provided written informed consent for the sample collection and following analysis, prior to inclusion in the study.

## Author contributions

ZZ and JPH conceived and designed the experiments. ZZ performed the experiments. WHM conducted rectal *E*. *coli* collection. RFP and GD conducted genome sequencing and alignments. JAW conducted mass spectrometry. ZZ and GLK conducted reporter construct and targeted mutagenesis. ZZ, PJM, and JPH conducted network analyses. ZZ and JPH analyzed the data. ZZ and JPH wrote the manuscript.

## Supplementary Material

Supplemental data

## Figures and Tables

**Table 1:**
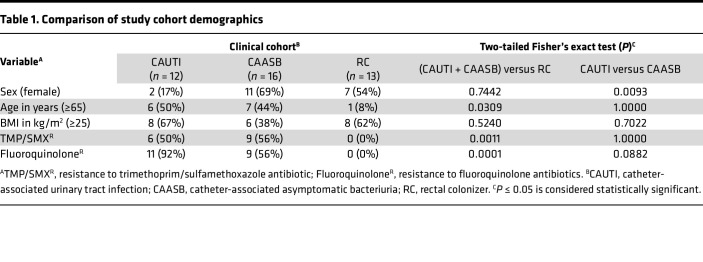
Comparison of study cohort demographics

**Table 2:**
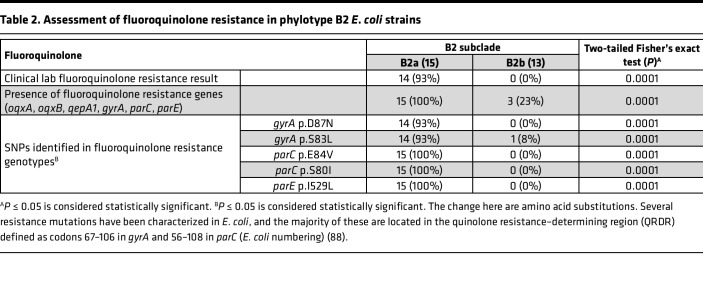
Assessment of fluoroquinolone resistance in phylotype B2 *E*. *coli* strains

**Table 3:**
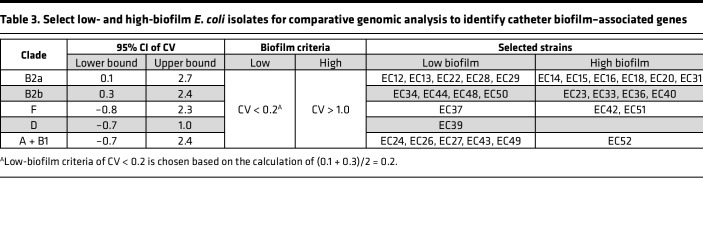
Select low- and high-biofilm *E*. *coli* isolates for comparative genomic analysis to identify catheter biofilm–associated genes

## Version 1 12/13/2022

In-Press Preview

## Version 2 01/24/2023

Electronic publication
