# Supplementary material for: E. coli catheter-associated urinary tract infections are associated with distinctive virulence and biofilm gene determinants
Source: JCI Insight. 2023 Jan 24;8(2):e161461. doi: 10.1172/jci.insight.161461 (PMC9977300; doi:10.1172/jci.insight.161461)
Supplement: Supplemental data [file jciinsight-8-161461-s079.pdf]

Escherichia coli catheter-associated urinary tract infections are associated with distinctive virulence and biofilm gene determinants

Supplementary information

Supplementary figures

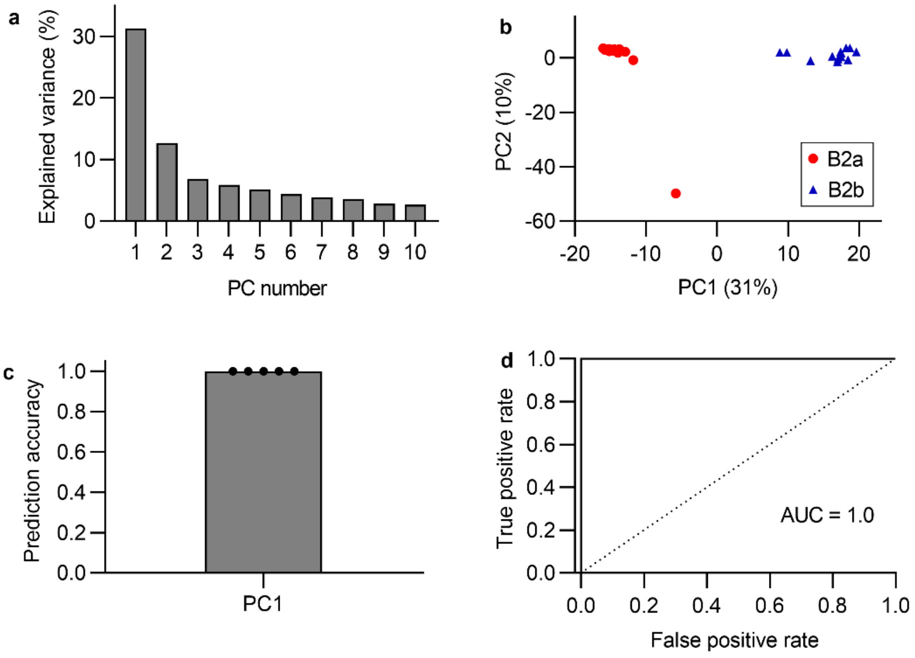

Supplementary Figure S1. Identification of two genetically different B2 subclades. (a) Explained variance of the first ten principal components in sparse principal component analysis (sPCA) of phylotype B2 strains gene composition. **(b)** Score plot of the first two principal components for displaying the group-wise clusterings between two B2 subclades, B2a and B2b. **(c)** Logistic regression using sPCA-derived PC1 values for classifying B2a and B2b yielded a prediction accuracy of 1.0 (SD = 0) with 5-fold cross validations. **(d)** Logistic regression using sPCA-derived PC1 values for classifying B2a and B2b yielded an AUC of 1.0 (SD = 0) with 5-fold cross validations.

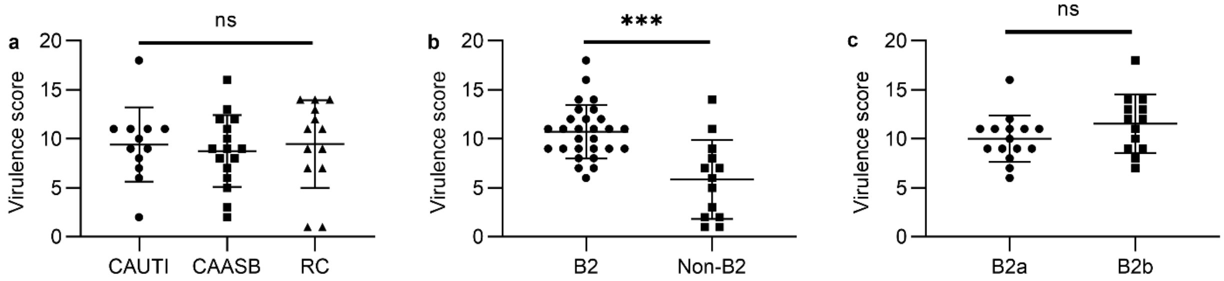

Supplementary Figure S2. Virulence scores in different phenotypic and genetic groups of *E. coli* strains. (a) Comparison of virulence scores between phenotypic groups, CAUTI vs CAASB vs RC. Mean with SD plotted for 12 CAUTI, 16 CAASB, and 13 RC strains, respectively. $P = 0.147$, by one-way ANOVA multiple comparisons test. **(b)** Comparison of virulence scores between phylotypic groups, B2 vs Non-B2. Mean with SD plotted for 28 B2 and 13 non-B2 strains, respectively. $P = 0.002$, by Mann-Whitney test. **(c)** Comparison of virulence score between subclades within phylotype B2 isolates, B2a vs B2b. Mean with SD plotted for 15 B2a and 13 B2b strains, respectively. $P = 0.138$, by Mann-Whitney test. $P <= 0.05$ is considered statistically significant. ns: not significant. ***: $P < 0.001$.

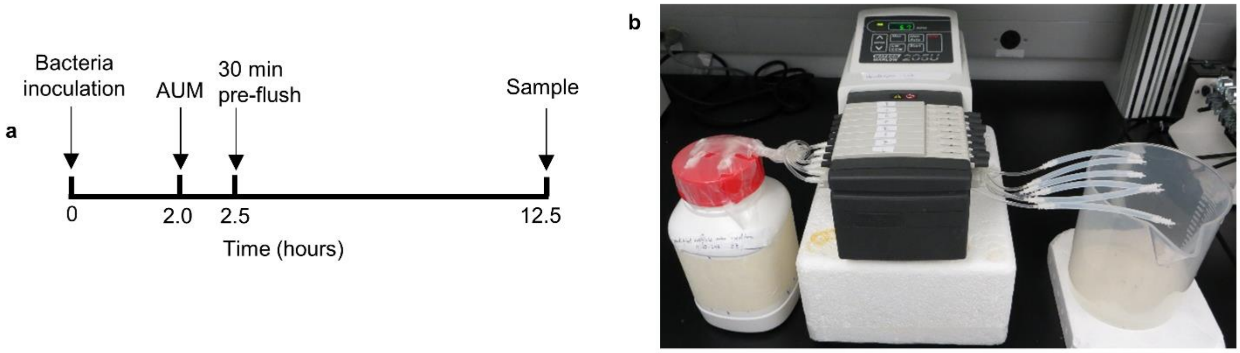

Supplementary Figure S3. Characterization of catheter-biofilm formation in a continuous flow model. (a) Experimental timeline of the biofilm formation test. **(b)** Schematic diagram of continuous flow catheter system, including influent, peristaltic pump, urinary catheter, and effluent. AUM: artificial urine medium (AUM).

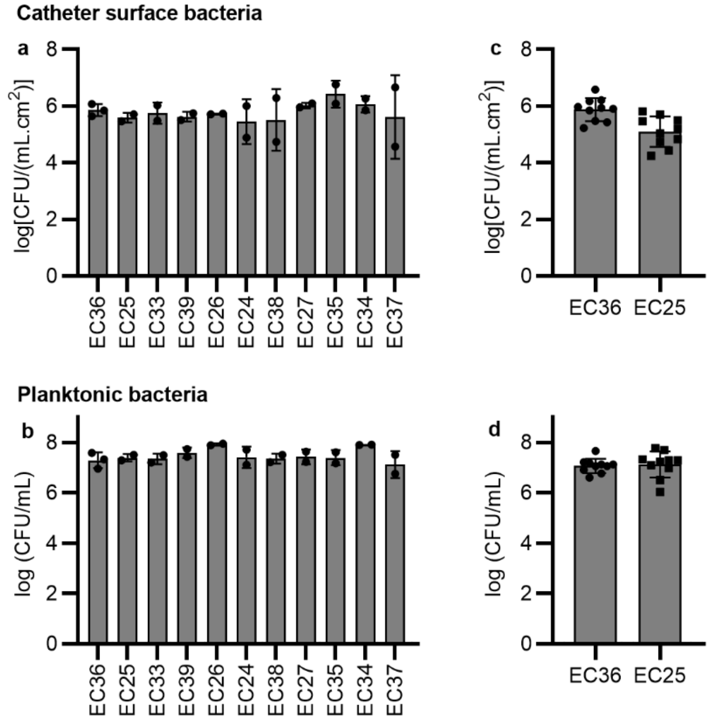

Supplementary Figure S4. Planktonic and biofilm bacteria growths in bacterial competition tests in catheter colonization model. (a & b) CFUs of ST131 CAUTI strain EC20 plus each of 11 non-ST131 CAASB strains (EC36, 25, 33, 39, 26, 24, 38, 27, 35, 34, and 37) in **(a)** catheter-adherent bacteria and **(b)** planktonic bacteria in pair-wise bacterial competition tests in the catheter colonization model. Three replicates with mean and SD plotted for EC36 and EC25, two replicates with mean and SD plotted for EC33, 39, 26, 24, 38, 27, 35, 34, and 37. **(c & d)** CFUs of each of 10 ST131 CAUTI strains (EC12, 13, 14, 15, 16, 17, 18, 19, 20, and 22) plus either of two non-ST131 CAASB strains (EC36 and EC25) in **(c)** catheter-adherent bacteria and **(d)** planktonic bacteria in pair-wise bacterial competition tests in the catheter colonization model. Ten ST131 CAUTI strains with mean and SD plotted.

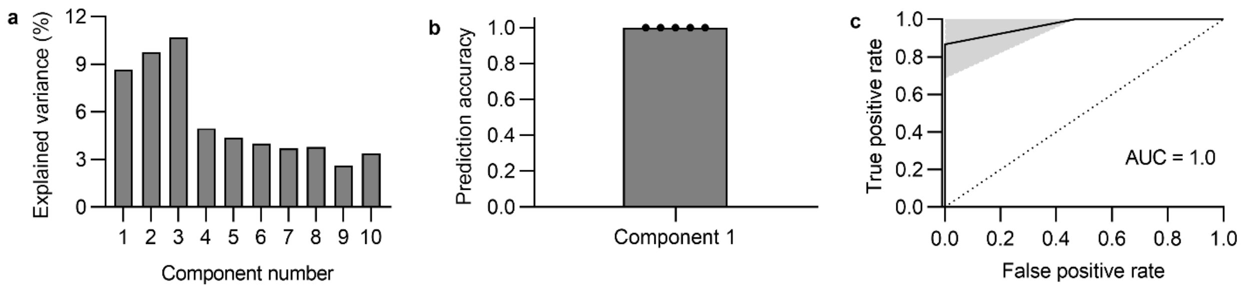

Supplementary Figure S5. Identification of catheter biofilm-associated genes. (a) Explained variance of the first ten principal components in sparse partial least squares discriminant analysis (sPLSDA) of gene composition of high and low biofilm *E. coli* strains. **(b)** Logistic regression using sPLSDA-derived PC1 values for classifying high and low biofilm strains yielded a prediction accuracy of 1.0 (SD = 0) with 5-fold cross validations. **(c)** Logistic regression using sPLSDA-derived PC1 values for classifying high and low biofilm strains yielded an AUC of 1.0 (SD = 0) with 5-fold cross validations.

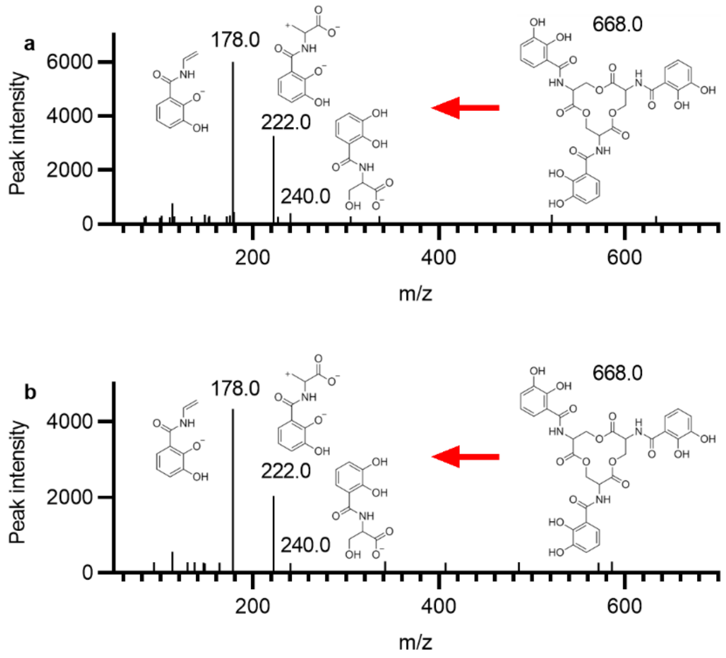

Supplementary Figure S6. Cyclic enterobactin is produced by both 52Δ*fecA* and 52Δ*fecA::fecA* during catheter-biofilm formation under continuous flow. (a) Tandem MS/MS product ion scan spectrum of cyclic enterobactin (m/z=-668.1) in the voided media of EC52Δ*fecA* continuous flow catheter-biofilm assay. **(b)** Tandem MS/MS product ion scan spectrum of cyclic enterobactin (m/z=-668.1) in the voided media of EC52Δ*fecA::fecA* continuous flow catheter-biofilm assay.

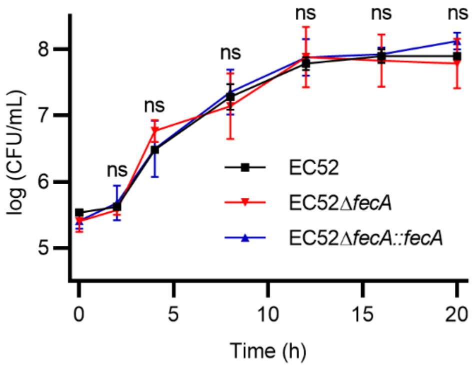

Supplementary Figure S7. Planktonic bacterial growth curves for EC52, EC52Δ*fecA*, and EC52Δ*fecA::fecA* strains. By one-way ANOVA with Dunnett's multiple comparisons test. $P <= 0.05$ is considered statistically significant. ns: not significant. *: $P <= 0.05$. **: $P < 0.01$. ***: $P < 0.001$. ****: $P < 0.0001$.

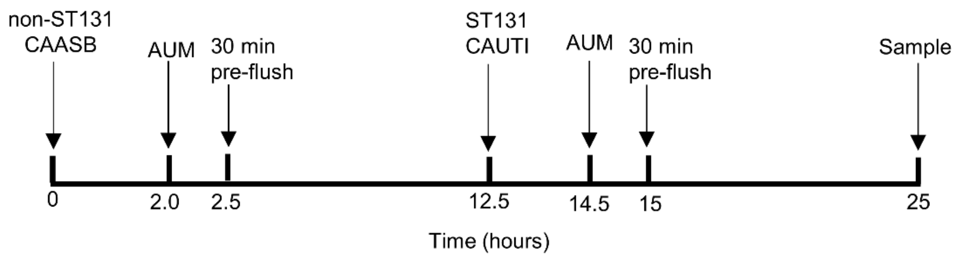

Supplementary Figure S8. Experimental timeline of bacterial interference test using continuous flow catheter model.

a

Seq_1 **GATCGTTGACCGTATCGTC**GGTCGCCGCGTTCATGCGCCGTCTGGTCGTGTTTATCACGT 60
  |#||||||||||||||#||#|||||||||||#||||||||||||||||||||||||||
Seq_2 --**TTGTTGACCGTATCGTAGGC**CGCCGCGTTCACGCGCCGTCTGGTCGTGTTTATCACGT 58

Seq_1 TAAATTCAATCCGCCGAAAGTAGAAGGCAAAGACGACGTTACCGGTGAAGAACTGACTAC 120
|||||||||||||||||||||||||||||||||||||||||||||||||||#||||||||
Seq_2 TAAATTCAATCCGCCGAAAGTAGAAGGCAAAGACGACGTTACCGGTGAAGAGCTGACTAC 118

Seq_1 CCGTAAAGA**TGATCAGGAAGAGACCGTAC** 149
|||||||||#||||||||||||||||||
Seq_2 CCGTAAAG**ACGATCAGGAAGAGACCGTA**- 146

b

Seq_1 -**TGTTGATCGTATCGTCGGT**CGCCGCGTTCACGCGCCATCTGGTCGTGTTTATCACGTTA 59
 ||||||#||||||||#||#|||||||||||||||||#||||||||||||||||||||||
Seq_2 **TTGTTGACCGTATCGTAGGC**CGCCGCGTTCACGCGCCGTCTGGTCGTGTTTATCACGTTA 60

Seq_1 AATTCAATCCGCCGAAAGTAGAAGGCAAAGACGACGTTACCGGTGAAGAACTGACTACCC 119
|||||||||||||||||||||||||||||||||||||||||||||||||#||||||||||
Seq_2 AATTCAATCCGCCGAAAGTAGAAGGCAAAGACGACGTTACCGGTGAAGAGCTGACTACCC 120

Seq_1 GTAAAGACGATCAGGAAGA**AACCGTGCGTAAACGTCT** 156
|||||||||||||||||||#|||||#
Seq_2 GTAAAG**ACGATCAGGAAGAGACCGTA**----------- 146

c

Seq_1 **TGACCGAGTTCGAATATGAC**ATTCTGGCGAAACGTCTGCGTGAGTTGTCGTTCCTCAACT 60
||||||||||||||||||#|||||||||||||||||||||||||||||||||||||||||
Seq_2 **TGACCGAGTTCGAATATGAA**ATTCTGGCGAAACGTCTGCGTGAGTTGTCGTTCCTCAACT 60

Seq_1 CCGGCGTTTCCATTCGTCTGCGCGACAAGCGCGATGGCAAAGAAGACCACTTCCACTATG 120
|||||||||||||||||||||||||#||||||||#|||||||||||||||||||||||||
Seq_2 CCGGCGTTTCCATTCGTCTGCGCGATAAGCGCGACGGCAAAGAAGACCACTTCCACTATG 120

Seq_1 AAGGCGGCATCAAGGCATTCGTTGAATATCTGAACAAGAACAAAACGCCGATCCACCCGA 180
||||||||||||||||#|||||||||||||||||||||||||||||||||||||||||||
Seq_2 AAGGCGGCATCAAGGCGTTCGTTGAATATCTGAACAAGAACAAAACGCCGATCCACCCGA 180

Seq_1 ATATCTTCTACTT**CTCCACCGAAAAAGACG** 210
|||||||||||||#||||||||||||||||
Seq_2 ATATCTTCTACTT**TTCCACCGAAAAAGACG** 210

d

Seq_1 **CTCAGGAAGACTGCCGTAAT**GGTTTCCTGTTGGACGGCTTCCCGCGTACCATTCCGCAGG 60
   |||||||||||||#||#||||||||||||||||||||||||||||||||||||||||
Seq_2 ---**AGGAAGACTGCCGCAAC**GGTTTCCTGTTGGACGGCTTCCCGCGTACCATTCCGCAGG 57

Seq_1 CAGACGC**GATGAAAGAAGCGGGCAT** 85
|||||||#|||||||||||||||||
Seq_2 CAGACGC**AATGAAAGAAGCGGGCAT** 82

Supplementary Figure S9. Gene sequences alignments for identifying SNPs-containing DNA segments that can differentiate the 11 non-ST131 CAASB from the 10 ST131 CAUTI isolates in a defined mixture of two bacteria. SNPs-containing DNA regions are labelled as

bold, with SNPs labelled as "#". **(a)** Gene *adk* (adenylate kinase) is identified with SNPs-containing regions that can differentiate EC24, EC25, and EC26 from 10 ST131 CAUTI isolates. Seq_1 = EC24, EC25, and EC26. Seq_2 = Ten ST131 CAUTI isolates. **(b)** Gene *adk* (adenylate kinase) is identified with SNPs-containing regions that can differentiate EC27 from 10 ST131 CAUTI isolates. Seq_1 = EC27. Seq_2 = Ten ST131 CAUTI isolates. **(c)** Gene *gyrB* (DNA gyrase) is identified with SNPs-containing regions that can differentiate EC33, EC34, EC35, and EC36 from 10 ST131 CAUTI isolates. Seq_1 = EC33, EC34, EC35, and EC36. Seq_2 = Ten ST131 CAUTI isolates. **(d)** Gene *adk* (adenylate kinase) is identified with SNPs-containing regions that can differentiate EC37, EC38, and EC39 from 10 ST131 CAUTI isolates. Seq_1 = EC37, EC38, and EC39. Seq_2 = Ten ST131 CAUTI isolates.

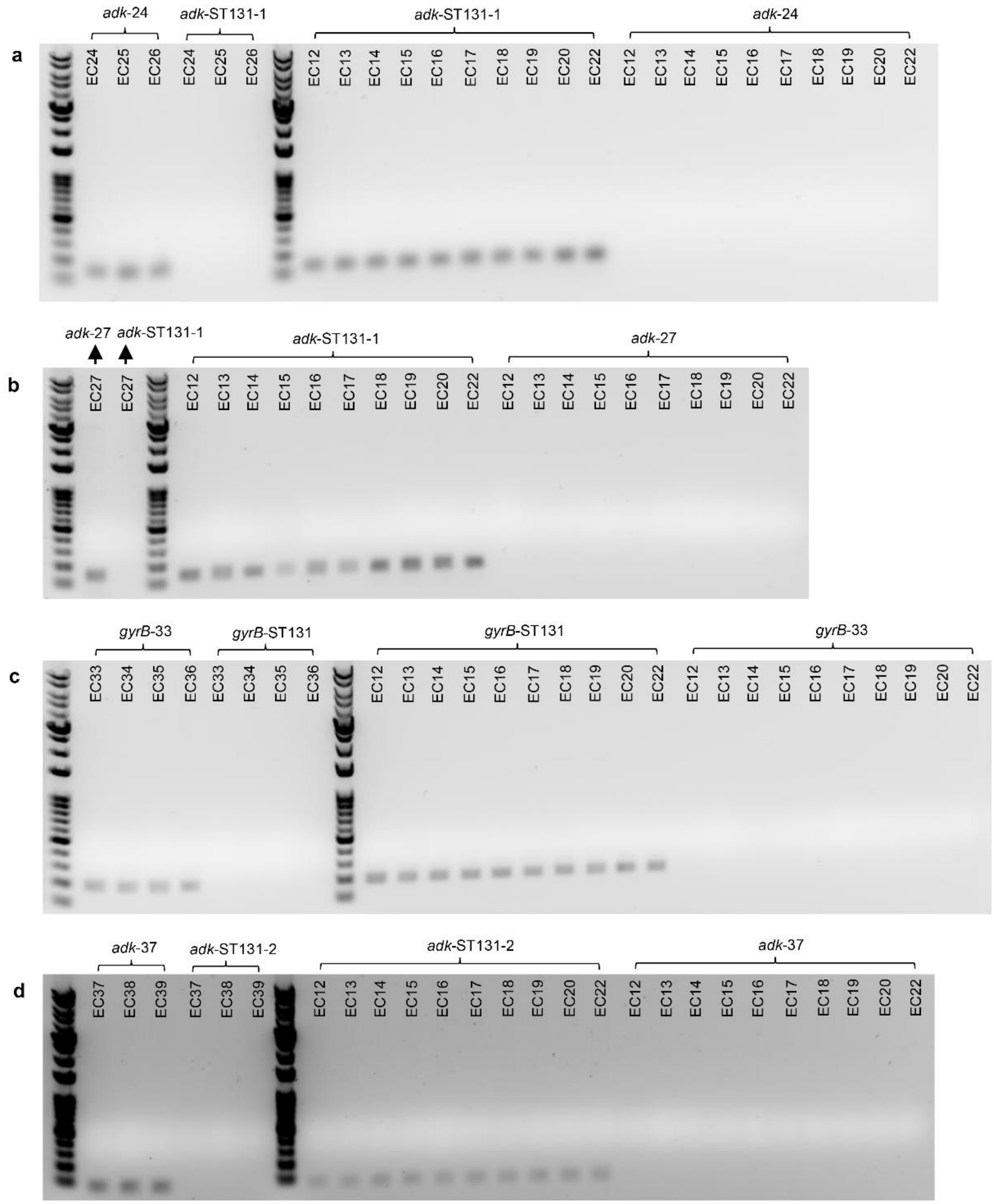

Supplementary Figure S10. Polymerase chain reaction (PCR) and gel electrophoresis validate that SNPs-based assays can distinguish two *E. coli* isolates in the mixed cultures of competition tests. (a) Primers *adk*-24 (forward & reverse) can only amplify specific SNPs-containing *adk* sequences of EC24, EC25, and EC26, but not of 10 ST131 CAUTI isolates. In contract, primers *adk*-ST131-1 (forward & reverse) can only amplify specific SNPs-containing *adk* sequences of 10 ST131 CAUTI isolates, but not of EC24, EC25, and EC26.

Therefore, the specific SNPs-containing *adk* sequences are able to distinguish EC24, EC25, and EC26 from the 10 ST131 CAUTI isolates. **(b)** Primers *adk*-27 (forward & reverse) can only amplify specific SNPs-containing *adk* sequences of EC27, but not of 10 ST131 CAUTI isolates. In contract, primers *adk*-ST131-1 (forward & reverse) can only amplify specific SNPs-containing *adk* sequences of 10 ST131 CAUTI isolates, but not of EC27. Therefore, the specific SNPs-containing *adk* sequences are able to distinguish EC27 from the 10 ST131 CAUTI isolates. **(c)** Primers *gyrB*-33 (forward & reverse) can only amplify specific SNPs-containing *gyrB* sequences of EC33, EC34, EC35, and EC36, but not of 10 ST131 CAUTI isolates. In contract, primers *gyrB*-ST131 (forward & reverse) can only amplify specific SNPs-containing *gyrB* sequences of 10 ST131 CAUTI isolates, but not of EC33, EC34, EC35, and EC36. Therefore, the specific SNPs-containing *gyrB* sequences are able to distinguish EC33, EC34, EC35, and EC36 from the 10 ST131 CAUTI isolates. **(d)** Primers *adk*-37 (forward & reverse) can only amplify specific SNPs-containing *adk* sequences of EC37, EC38, and EC39, but not of 10 ST131 CAUTI isolates. In contract, primers *adk*-ST131-2 (forward & reverse) can only amplify specific SNPs-containing *adk* sequences of 10 ST131 CAUTI isolates, but not of EC37, EC38, and EC39. Therefore, the specific SNPs-containing *adk* sequences are able to distinguish EC37, EC38, and EC39 from the 10 ST131 CAUTI isolates.

Supplementary tables

Supplementary Table S1. Pan-genome gene counts for 41 clinical *E. coli* isolates

***E. coli* isolate**	**Pan-genome size**
EC12	4763
EC13	4873
EC14	4754
EC15	4856
EC16	4789
EC17	4796
EC18	4701
EC19	4743
EC20	4658
EC21	4320
EC22	4578
EC23	4813
EC24	4787
EC25	4716
EC26	4460
EC27	4579
EC28	5088
EC29	5983
EC30	4745
EC31	4656
EC32	4866
EC33	4795
EC34	4776
EC35	4627
EC36	4856
EC37	4568
EC38	4829
EC39	4326
EC40	4612
EC41	4901
EC42	5078
EC43	4660
EC44	4749
EC46	4864
EC47	4618
EC48	4645
EC49	4662
EC50	4629
EC51	4879
EC52	4919

EC53	4770

Supplementary Table S2. Pan-genome gene counts for 15 B2a sub-clade *E. coli* isolates

B2a isolate	Pan-genome size
EC12	4763
EC13	4873
EC14	4754
EC15	4856
EC16	4789
EC17	4796
EC18	4701
EC19	4743
EC20	4658
EC22	4578
EC28	5088
EC29	5983
EC30	4745
EC31	4656
EC32	4866

Supplementary Table S3. Carriage of antibiotic resistance genes in phylotype B2 *E. coli* strains.

Antibiotic	Antibiotic resistance gene	B2 subclade		Two-tailed Fisher's exact test (P)[a]
		B2a (15)	B2b (13)	
Aminoglycoside	*aac(3)-IId*, *aac(6')-Ib-cr*, *aac(6')-Ib3*, *aadA1, aadA2*, *aadA5*, *aph(3'')-Ib, aph(3')-Ia*, *aph(6)-Id, strA*	11 (73%)	2 (15%)	0.0032
Beta-lactam	*blaCMY-2*, *blaCMY-7*, *blaLEN16*, *blaOXA-1*, *blaTEM-1B*, *blaTEM-1C*, *ampC*	15 (100%)	6 (46%)	0.0014
Amphenicol	*catA1*, *catA2*, *catB4*, *cmlA1*	3 (20%)	0 (0%)	0.2262
TMP/SMX	*dfrA1*, *dfrA12*, *dfrA15*, *dfrA17*, *dfrA19*, *dfrA5*, *dfrB4*, *sul1*, *sul2*, *sul3*	9 (60%)	2 (15%)	0.0238
MLS	*erm(B)*, *mdf(A)*, *mph(A)*	15 (100%)	12 (92%)	0.4643
Fluoroquinolone	*oqxA*, *oqxB*, *qepA1*, *gyrA*, *parC*, *parE*	15 (100%)	3 (23%)	0.0001
Tetracycline	*tet(A)*, *tet(B)*, *tet(D)*, *tet(M)*	7 (47%)	2 (15%)	0.1145

[a] $P <= 0.05$ is considered statistically significant.

Supplementary Table S4. Seventy-two biofilm-associated genes.

**Biofilm level**	**Biofilm-associated genes**
High-biofilm (46)	*fecA*, *fecB*, *fecC*, *fecD*, *fecE*, *fecI*, *fecR*, *iucA*, *iucB*, *iucC*, *iucD*, *iutA*, *group_2401*, *group_1339*, *ltrA*, *fucP*, *group_5022*, *group_6794*, *group_1842*, *ssuA*, *group_2483*, *group_2486*, *iraM*, *group_2393*, *group_6792*, *group_6793*, *group_6910*, *rbsK*, *group_1398*, *group_163*, *group_3360*, *group_523*, *group_6919*, *group_990*, *adrB*, *cirA*, *group_21*, *group_6911*, *group_5101*, *ccdB*, *group_1036*, *group_2994*, *ylpA*, *flu*, *group_193*, *group_246*
Low-biofilm (26)	*group_7558*, *group_1149*, *group_1713*, *group_228*, *group_312*, *tfaE*, *ascG*, *bglH*, *group_2755*, *tufB*, *gnu*, *group_3170*, *mbtM*, *group_1780*, *hicB*, *rhsB*, *tap*, *yedR*, *group_1225*, *group_1438*, *group_2524*, *group_3689*, *group_405*, *group_5064*, *group_5299*, *group_7629*

Supplementary Table S5. Recipe of the artificial urine medium (AUM)

Component	Quantify (g)	Concentration (mmol/L)
Oxoid Peptone L37	1	/
Yeast extract	0.005	/
Lactic acid	0.1	1.1
Citric acid	0.4	2
Sodium bicarbonate	2.1	25
Urea	10	170
Uric acid	0.07	0.4
Creatinine	0.8	7
Calcium chloride.2H_2O	0.37	2.5
Sodium chloride	5.2	90
Zinc sulfate.7 H_2O	0.00209	0.007
Magnesium sulfate.7H_2O	0.49	2
Sodium sulfate.10H_2O	3.2	10
Potassium dihydrogen phosphate	0.95	7
Di-potassium hydrogen phosphate	1.2	7
Ammonium chloride	1.3	25
Distilled water	To 1 Liter	

Supplementary Table S6. Eleven non-ST131 CAASB and Ten ST131 CAUTI *E. coli* isolates selected for bacterial interference tests.

Group	Strain	Phylotype	Sequence type (ST)
ST131 CAUTI (10)	EC12	B2	131
	EC13	B2	131
	EC14	B2	131
	EC15	B2	131
	EC16	B2	131
	EC17	B2	131
	EC18	B2	131
	EC19	B2	131
	EC20	B2	131
	EC22	B2	131
Non-ST131 CAASB (11)	EC24	B1	10
	EC25	B1	167
	EC26	B1	744
	EC27	B1	Untypeable
	EC33	B2	144
	EC34	B2	95
	EC35	B2	12
	EC36	B2	12
	EC37	D	354
	EC38	D	68
	EC39	D	1884

Supplementary Table S7. SNPs-qPCR standard curves for differentially quantifying non-ST131 CAASB and ST131 CAUTI *E. coli* DNAs in mixed bacteria cultures from bacterial interference tests.

***log(Cq)* = A*log(DNA)* + B[a]**								
***adk*[b]**			***gyrB*[c]**			***adk*[d]**		
Non-ST131 CAASB	EC24	y = -0.0841x + 1.2150	Non-ST131 CAASB	EC33	y = -0.0775x + 1.1971	Non-ST131 CAASB	EC37	y = -0.0808x + 1.2399
	EC25	y = -0.0796x + 1.2055		EC34	y = -0.0751x + 1.2819		EC38	y = -0.0746x + 1.2921
	EC26	y = -0.0837x + 1.2036		EC35	y = -0.0904x + 1.1820		EC39	y = -0.0738x + 1.2464
	EC27	y = -0.0872x + 1.1886		EC36	y = -0.0824x + 1.1836	ST131 CAUTI	EC12	y = -0.0751x + 1.2525
ST131 CAUTI	EC12	y = -0.0803x + 1.2313	ST131 CAUTI	EC12	y = -0.0859x + 1.2264		EC13	y = -0.0765x + 1.2707
	EC13	y = -0.0776x + 1.2508		EC13	y = -0.0795x + 1.2450		EC14	y = -0.0792x + 1.2323
	EC14	y = -0.0773x + 1.2168		EC14	y = -0.0836x + 1.2132		EC15	y = -0.0793x + 1.2225
	EC15	y = -0.0777x + 1.2006		EC15	y = -0.0759x + 1.2005		EC16	y = -0.0805x + 1.2153
	EC16	y = -0.0817x + 1.2068		EC16	y = -0.0706x + 1.2179		EC17	y = -0.0747x + 1.2535
	EC17	y = -0.0800x + 1.2331		EC17	y = -0.0831x + 1.2230		EC18	y = -0.0688x + 1.2639
	EC18	y = -0.0749x + 1.2186		EC18	y = -0.0751x + 1.2201		EC19	y = -0.0724x + 1.2664
	EC19	y = -0.0800x + 1.2442		EC19	y = -0.0785x + 1.2393		EC20	y = -0.0810x + 1.1940
	EC20	y = -0.0914x + 1.1798		EC20	y = -0.0838x + 1.1845		EC22	y = -0.0681x + 1.2778
	EC22	y = -0.0747x + 1.2551		EC22	y = -0.0812x + 1.2494			

[a] Standard curve for quantifying *E. coli* DNAs in mixed bacterial culture from bacterial interference tests. *Cq*: threshold cycles in qPCR. DNA: concentration of DNA, ng/µL.

[b] SNPs identified in one portion of gene *adk* (adenylate kinase) are able to differentiate non-ST131 CAASB strains, EC24, EC25, EC26, and EC27, from ten ST131 CAUTI strains.

[c] SNPs identified in one portion of gene *gyrB* (DNA gyrase) are able to differentiate non-ST131 CAASB strains, EC33, EC34, EC35, and EC36, from ten ST131 CAUTI strains.

[d] SNPs identified in another portion of gene *adk* (adenylate kinase) are able to differentiate non-ST131 CAASB strains, EC37, EC38, EC39, and EC27, from ten ST131 CAUTI strains.

Supplementary Table S8. Primers used in this study

Primer Number	Name	Sequence (5'-3')	Description
ZZ#12	pGK73-Conf FP	TGAACACCATAACCGAAAGTAGT	pGK73 plasmid confirmation forward primer
ZZ#13	pGK73-Conf RP	ACCTTGAAGCGCATGAACT	pGK73 plasmid confirmation reverse primer
ZZ#14	fecI-pGK73-Cons FP	GATCGAGCTCCATCTGATGGAAATGGAAGCCAC	fecI-pGK73 plasmid construct forward primer
ZZ#15	fecI-pGK73-Cons RP	GATCGGATCCCATGCGGAGTGCATCAAAAGTTA	fecI-pGK73 plasmid construct reverse primer
ZZ#16	fecI-pGK73-Conf FP	TGAACACCATAACCGAAAGTAGT	fecI-pGK73 plasmid confirmation forward primer
ZZ#17	fecI-pGK73-Conf RP	TTATTGACATCCTCACTGCCC	fecI-pGK73 plasmid confirmation reverse primer
ZZ#18	fecA-KO FP	TTCTCGTTCGACTCATAGCTGAACACAACAAAAATGATGATGGGGAAGGTATTGTGTAGGCTGGAGCTGC	Red recombinase EC52 fecA knock out forward primer
ZZ#19	fecA-KO RP	CAACATAATCACATTCCAGCTAAAAGCCCGGCAAGCCGGGCGTTAACACAGGTCCATATGAATATCCTCCTTAGTTC	Red recombinase EC52 fecA knock out reverse primer
ZZ#20	fecA-Conf FP	GCGGTACTGGATAAACATTTCAC	EC52 fecA knock out confirmation forward primer
ZZ#21	fecA-Conf RP	CAGGCCTGCAAAAAGAAAAC	EC52 fecA knock out confirmation reverse primer
ZZ#22	fecA-pKT25-Cons FP	GACTAAGCTTGGAAAATAATTCTTATTTCGATTG	fecA-pKT25 plasmid construct forward primer
ZZ#23	fecA-pKT25-Cons RP	GACTGAATTCTCAGAACTTCAACGACCCCTGC	fecA-pKT25 plasmid construct reverse primer
ZZ#24	fecA-pKT25-Conf FP	ATCACATATTCTGCTGACGCA	fecA-pKT25 plasmid confirmation forward primer
ZZ#25	fecA-pKT25-Conf RP	GTTTTACCTGCAGTCCGCTG	fecA-pKT25 plasmid confirmation reverse primer
ZZ#26	adk-24 FP	GATCGTTGACCGTATCGTC	qPCR of adk SNPs-containing region in EC24, EC25, and EC26 forward primer
ZZ#27	adk-24 RP	GTACGGTCTCTTCCTGATCA	qPCR of adk SNPs-containing region in EC24, EC25, and EC26 reverse primer
ZZ#28	adk-27 FP	TGTTGATCGTATCGTCGGT	qPCR of adk SNPs-containing region in EC27 forward primer
ZZ#29	adk-27 RP	AGACGTTTACGCACGGTT	qPCR of adk SNPs-containing region in EC27 reverse primer
ZZ#30	adk-ST131-1 FP	TTGTTGACCGTATCGTAGGC	qPCR of adk SNPs-containing region-1 in ST131-CAUTI isolates forward primer
ZZ#31	adk-ST131-1 RP	TACGGTCTCTTCCTGATCGT	qPCR of adk SNPs-containing region-1 in ST131-CAUTI isolates reverse primer
ZZ#32	gyrB-33 FP	TGACCGAGTTCGAATATGAC	qPCR of gyrB SNPs-containing region in EC33, EC34, EC35, and EC36 forward primer
ZZ#33	gyrB-33 RP	CGTCTTTTTCGGTGGAG	qPCR of gyrB SNPs-containing region in EC33, EC34, EC35, and EC36 reverse primer
ZZ#34	gyrB-ST131 FP	TGACCGAGTTCGAATATGAA	qPCR of gyrB SNPs-containing region in ST131-CAUTI isolates forward primer
ZZ#35	gyrB-ST131 RP	CGTCTTTTTCGGTGGAA	qPCR of gyrB SNPs-containing region in ST131-CAUTI isolates reverse primer
ZZ#36	adk-37 FP	CTCAGGAAGACTGCCGTAAT	qPCR of adk SNPs-containing region in EC37, EC38, and EC39 forward primer
ZZ#37	adk-37 RP	ATGCCCGCTTCTTTCATC	qPCR of adk SNPs-containing region in EC37, EC38, and EC39 reverse primer
ZZ#38	adk-ST131-2 FP	AGGAAGACTGCCGCAAC	qPCR of adk SNPs-containing region-2 in ST131-CAUTI isolates forward primer
ZZ#39	adk-ST131-2 RP	ATGCCCGCTTCTTTCATT	qPCR of adk SNPs-containing region-2 in ST131-CAUTI isolates reverse primer

Supplementary Table S9. Strains derived for this study to assess *fec* expression and *fec* mutant biofilm formation.

Strain	Relevant antibiotic resistance[a]	Characteristic	Reference
EC52		Wild type *E. coli* rectal colonization isolate, high-biofilm former	[1, 2]
EC52::RFP	Amp^R	EC52 ectopically expressing RFP from pGK73 plasmid	This study
EC52::*fecI*-RFP	Amp^R	EC52 ectopically expressing RFP from *fecI*-pGK73 plasmid	This study
EC52Δ*fecA*		EC52 with an in-frame deletion of *fecA*, ferric citrate transport deficient	This study
EC52Δ*fecA*::*fecA*	Kan^R	EC52Δ*fecA* with an ectopic *fecA* complementation from *fecA*-pKT25 plasmid	This study

[a] Amp^R, resistance to ampicillin antibiotic. Kan^R, resistance to kanamycin antibiotic.

References

1. Marschall J, Zhang L, Foxman B, Warren DK, Henderson JP, Program CDCPE. Both host and pathogen factors predispose to Escherichia coli urinary-source bacteremia in hospitalized patients. Clin Infect Dis. 2012;54(12):1692-8. Epub 2012/03/21. doi: 10.1093/cid/cis252. PubMed PMID: 22431806; PubMed Central PMCID: PMCPMC3357479.
2. Jonas Marschall MLP, Betsy Foxman, Lixin Zhang, David K Warrenj, Jeffrey P Henderson. Patient characteristics but not virulence factors discriminate between asymptomatic and symptomatic. BMC Infectious Diseases. 2013;13(213).
